# Characterization of primary cilia during the differentiation of retinal ganglion cells in the zebrafish

**DOI:** 10.1186/s13064-016-0064-z

**Published:** 2016-04-06

**Authors:** Paola Lepanto, Camila Davison, Gabriela Casanova, Jose L. Badano, Flavio R. Zolessi

**Affiliations:** Human Molecular Genetics Laboratory, Institut Pasteur de Montevideo, Mataojo 2020, Montevideo, 11400 Uruguay; Cell Biology of Neural Development Laboratory, Institut Pasteur de Montevideo, Mataojo 2020, Montevideo, 11400 Uruguay; Sección Biología Celular, Departamento de Biología Celular y Molecular, Facultad de Ciencias, Universidad de la República, Iguá 4225, Montevideo, 11400 Uruguay; Unidad de Microscopía Electrónica, Facultad de Ciencias, Universidad de la República, Iguá 4225, Montevideo, 11400 Uruguay

**Keywords:** Retina, Cilia, Retinal ganglion cell, Neurogenesis

## Abstract

**Background:**

Retinal ganglion cell (RGC) differentiation in vivo is a highly stereotyped process, likely resulting from the interaction of cell type-specific transcription factors and tissue-derived signaling factors. The primary cilium, as a signaling hub in the cell, may have a role during this process but its presence and localization during RGC generation, and its contribution to the process of cell differentiation, have not been previously assessed in vivo.

**Methods:**

In this work we analyzed the distribution of primary cilia in vivo using laser scanning confocal microscopy, as well as their main ultrastructural features by transmission electron microscopy, in the early stages of retinal histogenesis in the zebrafish, around the time of RGC generation and initial differentiation. In addition, we knocked-down *ift88* and *elipsa*, two genes with an essential role in cilia generation and maintenance, a treatment that caused a general reduction in organelle size. The effect on retinal development and RGC differentiation was assessed by confocal microscopy of transgenic or immunolabeled embryos.

**Results:**

Our results show that retinal neuroepithelial cells have an apically-localized primary cilium usually protruding from the apical membrane. We also found a small proportion of sub-apical cilia, before and during the neurogenic period. This organelle was also present in an apical position in neuroblasts during apical process retraction and dendritogenesis, although between these stages cilia appeared highly dynamic regarding both presence and position. Disruption of cilia caused a decrease in the proliferation of retinal progenitors and a reduction of neural retina volume. In addition, retinal histogenesis was globally delayed albeit RGC layer formation was preferentially reduced with respect to the amacrine and photoreceptor cell layers.

**Conclusions:**

These results indicate that primary cilia exhibit a highly dynamic behavior during early retinal differentiation, and that they are required for the proliferation and survival of retinal progenitors, as well as for neuronal generation, particularly of RGCs.

**Electronic supplementary material:**

The online version of this article (doi:10.1186/s13064-016-0064-z) contains supplementary material, which is available to authorized users.

## Background

Developmental processes are carried out based on a complex interaction between information inherited from the parent cell and time/tissue-specific environmental cues. The vertebrate retina is one of the most organized tissues in the body. To achieve this unique organization, dividing neuroepithelial cells must give rise to differentiating neuroblasts in a highly controlled and orderly fashion. Even though retinal ganglion cells (RGCs) are the first neuroblasts to be born, how these cells arise and differentiate into the correct neuronal type, with its corresponding morphology and connections, is still not completely understood.

Both cell type-specific expression of transcription factors and tissue-derived positional and trophic factors are likely to interact to achieve a mature and fully functional retina. Several signals from the environment have been shown to influence cell position and differentiation. For example, Notch signaling in relation to interkinetic nuclear migration has been linked to cell cycle withdrawal, the first step in the differentiation process [1]. The basal lamina of the neuroepithelium (the “inner limiting membrane”) has also been shown to play a critical role as RGC axon extension and orientation depends on the presence of Laminin [2]. Another contributing signaling molecule is Sonic Hedgehog (Shh), which is needed for spreading the wave of RGC and amacrine cell differentiation across the retina, as well as later on for photoreceptor cell differentiation [3–7]. Importantly, during RGC differentiation in vivo, neuroepithelial polarity is transiently maintained: it has been shown that polarity determinants are apically-positioned during the initial stages of differentiation, while in vitro these determinants show an erratic behavior [8]. Therefore, the tissue impinges constraints on the inherited differentiation program, guiding it to achieve the mature functional structure.

In recent years it has been shown that one of the main signaling hubs in cells, which has a critical role during development, is the primary cilium. Primary cilia are microtubule-based organelles that extend from a modified centriole, the basal body, protruding as an extension of the plasma membrane. Cilia are enriched in moieties required for sensing and transducing a number of signaling cascades that have been shown to rely on this particular cellular structure, including Wnt and Shh [9]. These findings, coupled with the ubiquitous presence of primary cilia in different cell types, explain why defects in the formation, maintenance and function of this organelle result in a range of clinical manifestations that have been grouped under the term ciliopathies [10, 11]. Importantly, central nervous system associated phenotypes, including structural defects, mental retardation and retinal degeneration, are hallmark phenotypes of several ciliopathies [12].

Primary cilia have been studied in the context of neuronal differentiation in different regions of the central nervous system. It has been shown for example that this organelle plays a role in progenitor cell proliferation in the cerebellum [13, 14], proliferation of progenitors and integration of neurons in the hippocampus [15, 16], and in the migration of neuroblasts in the mouse developing cortex [17, 18]. Early electron microscopy studies have shown that both neural tube and retinal neuroepithelial cells have an apically localized primary cilium, and that RGC neuroblasts of the mouse developing retina have a primary cilium that also displays a polarized localization, being positioned at the tip of the retracting apical process [19, 20]. Therefore, it is possible that the primary cilium is playing a role in RGC differentiation.

In this work we performed an in-depth characterization of the presence and localization of cilia during the differentiation of RGCs in the zebrafish retina combining electron and confocal microscopy with time-lapse video microscopy in live embryos. As an in vivo marker for cilia, we used a zebrafish transgenic line expressing EGFP fused to the carboxy-terminus of the small GTPase Arl13b (Arl13b-GFP; [21]). Arl13b, which belongs to the Arl/Arf family of GTPases involved in microtubule dynamics and membrane traffic, is specifically localized to the ciliary axoneme and is an essential protein for cilia maintenance in zebrafish and mice [22, 23]. In addition, we evaluated retinal development in conditions where cilia integrity was compromised. Our data show that RGCs primary cilia are highly dynamic organelles, changing in size and position during the differentiation process. The double knockdown of *ift88* and *elipsa*, two genes important for cilia formation and maintenance, shows that this organelle plays a role both during progenitor cell proliferation and maintenance, as well as during neurogenesis. Thus, our data provide important information that will help in achieving a more complete understanding of the role of primary cilia in RGC differentiation.

## Methods

### Fish breeding and care

Zebrafish were maintained and bred in a stand-alone system (Tecniplast), with controlled temperature (28 °C), conductivity (500 μS/cm2) and pH (7.5), under live and pellet dietary regime. Embryos were raised at temperatures ranging from 28.5 to 32 °C and staged in hours post-fertilization (hpf) according to Kimmel and collaborators [24]. We used wild-type (SAT; [25]) and different previously established transgenic lines in this work: Tg(actb2:Arl13b-GFP)hsc5 (Arl13b-GFP, kindly provided by B. Ciruna; [21]), Tg(atoh7:gap43-EGFP)cu1 (atoh7:gap-GFP; [8]), Tg(atoh7:gap43-RFP)cu2 (atoh7:gap-RFP; [8]), SoFa1 (atoh7:gap-RFP/ptf1a:cytGFP/crx:gap-CFP; [26]). In addition, we generated a double transgenic line crossing atoh7:gap-RFP and Arl13b-GFP. All the manipulations were carried out following the approved local regulations (CEUA-Institut Pasteur de Montevideo, and CNEA).

### Morpholino treatment

The morpholino oligomers (MOs) used in this study were obtained from Gene Tools (Philomath, USA) and included those previously used to target zebrafish *elipsa* and *ift88* translational initiation: *elipsa*-ATG (GGCTACCGATTCGTTCATGGCATCA; [27]) and *ift88*-ATG (GCCTTATTAAACAGAAATACTCCCA; IFT88 MO3, [28]). We also used newly designed morpholinos to target the splicing of *ift88* and *elipsa* mRNA: *ift88*-SP (AACAGCAGATGCAAAATGACTCACT) which targets the exon 3 - intron 3 boundary; *elipsa-*SP (CTGTTTTAATAACTCACCTCGCTGA) which targets the exon 1 - intron 1 boundary. All MOs were injected in the yolk of 1–4 cell-stage embryos, at a maximum volume of 4 nL. As control, we used matching doses of a standard MO (CCTCTTACCTCAGTTACAATTTATA) from Gene Tools (Philomath, USA). When considered necessary, we co-injected a double amount per embryo of the standard anti-p53 MO [29].

To test the effectiveness of splice-blocking morpholinos we performed RT-PCR (primers sequences are available upon request). Total RNA was extracted from 30 morphant or wild-type embryos using TRIzol reagent (Invitrogen) and cDNA was prepared using the SuperScript First-Strand Synthesis System for RT-PCR (Invitrogen).

### Blastomere transplantation

Blastula stage embryos were used for transplantation experiments. Cells from the donor embryos were transplanted into the animal pole of hosts, following standard procedures. After transplantation embryos were incubated in E3 medium (5 mM NaCl, 0.17 mM KCl, 0.33 mM CaCl_2_, 0.33 mM MgSO_4_) plus 10 mM Hepes pH 7.4, 0.00005 % methylene blue and Penicillin/Streptomycin (Sigma) at 32 °C.

### Immunofluorescence

Embryos were grown in 0.003 % phenylthiourea (Sigma) from 10 hpf onwards to delay pigmentation, and fixed overnight at 4 °C, by immersion in 4 % paraformaldehyde in phosphate saline buffer (PBS; pH 7.4).

For whole-mount immunostaining all subsequent washes were performed in PBS containing 1 % Triton X-100. Further permeability was achieved by incubating the embryos in 0.25 % trypsin-EDTA for 10–15 min at 0 °C. Blocking was for 30 min in 1 % bovine serum albumin (BSA), 1 % Triton X-100 in PBS. The primary antibodies, diluted in the blocking solution, were used as follows: zn8 (ZIRC, Oregon), 1/100; anti-activated Caspase 3 (AbCam), 1/500; anti-histone H3 pSer10 (Santa Cruz), 1/300, anti-acetylated tubulin (Sigma), 1/750; anti-γ tubulin (Sigma), 1/500. The secondary antibodies used were: anti-rabbit IgG-TRITC (Life Technologies), 1/1000; anti-mouse IgG-Alexa 488 (Life Technologies), 1/1000; anti-mouse IgG1-Alexa 488 (Life Technologies), 1/1000; anti-mouse IgG2b-Alexa 568 (Life Technologies), 1/1000. When necessary, TRITC-conjugated phalloidin (Sigma) was mixed with the secondary antibody. Nuclei were fluorescently counterstained with methyl green [30]. All antibody incubations were performed overnight at 4 °C. Embryos were mounted in 70 % glycerol in 20 mM Tris buffer (pH 8.0) and stored at 4 °C or −20 °C.

Five day-old embryos were fixed as described above, washed in PBS and cryoprotected in 30 % sucrose in PBS overnight at 4 °C. They were then embedded in OCT (Tissue-Tek) and quickly frozen in liquid N_2_. Transverse cryosections (10 μm) were made on a Reichert-Jung Cryocut E cryostat and adhered to gelatin subbed slides. Mounting was made using 70 % glycerol in 20 mM Tris buffer (pH 8.0).

Observation of whole embryos or cryosections was performed using a Leica TCS-SP5 (for all in vivo and some fixed material imaging) or a Zeiss LSM800 (for some fixed material imaging) laser confocal microscopes, with 63x 1.4 NA oil immersion or 20x 0.7 NA and 63x 1.3 NA glycerol:water (80:20) or water immersion objectives.

### In vivo confocal microscopy

Around 30 hpf embryos were selected, anesthetized using 0.04 mg/mL MS222 (Sigma) and mounted in 0.8 % low melting-point agarose (Sigma) over n° 0 glass bottom dishes (MaTek). After agarose solidification and during overnight image acquisitions, embryos were kept in Ringer’s solution (116 mM NaCl, 2.9 mM KCl, 1.8 mM CaCl_2_, 5 mM HEPES pH 7.2) with 0.04 mg/mL MS222. Live acquisitions were made using a Leica TCS-SP5 laser confocal microscope with a 20x 0.7 NA objective and glycerol:water (80:20) immersion medium. Stacks around 60 μm-thick were acquired in bidirectional mode, at 1 μm spacing and 512 × 512 pixel resolution every 10 or 15 min (the acquisition time for each embryo was approximately 45 s).

### Transmission electron microscopy

Embryos were fixed at 26, 35 and 48 hpf by immersion in fixative solution (4 % paraformaldehyde, 2.5 % glutaraldehyde in PBS, pH 7.2–7.4). Then the head was dissected and incubated overnight at 4 °C. The fixed material was washed in PBS, post-fixed in 1 % osmium tetroxide in distilled water for 1 h, and washed in distilled water. Samples were dehydrated through a graded (25, 50, 75, 95, 100 %) ethanol-water series, transferred to acetone for 2 × 20 min, and infiltrated with araldite resin through a series of steps (2:1 acetone:araldite for 30 min, 1:1 acetone:araldite for 30 min, 1:2 acetone:araldite for 30 min, 100 % araldite overnight at 4 °C). On the next day, the material was transferred into flat-embedding molds with freshly made araldite resin and oriented as desired. The embedded samples were cured at 60 °C for 48 h. Blocks were semi-thin sectioned at 500 nm using a RMC MT-X ultramicrotome, stained with boraxic methylene blue and examined in a light microscope. Once the area of interest was reached, ultrathin 70 nm sections were obtained and mounted on formvar-coated copper grids. Sections were stained for 2 h in 2 % aqueous uranyl acetate followed by staining in Reynold’s lead citrate for 10 min. Observation and acquisition was performed using a Jeol JEM 1010 transmission electron microscope operated at 100 kV, equipped with a Hamamatsu C4742-95 digital camera.

### Image analysis

Images were analyzed using Fiji [31]. Primary cilia length in Kupffer’s vesicle was measured manually in maximum intensity z-projections of the confocal stacks. Volume measurements (whole retina and zn8-positive region) were performed using intensity-based thresholding aided by manual selection. After that, the volume was obtained using 3D Roi Manager and 3D Objects Counter plugins [32, 33]. Cell counting (pH3 and activated Caspase 3-positive cells) was also done manually on the confocal stacks with the aid of the MTrackJ plugin [34]. For the fluorescence profile analysis of the retina in SoFa transgenic embryos, confocal planes from a 10 μm-deep stack were projected using intensity average and then a 100 μm-long and 20 μm-wide line selection was drawn across the retina. The fluorescence intensity along this line was measured for each channel using the Intensity Toolset from Imperial College of London FILM facility (http://www.imperial.ac.uk/medicine/facility-for-imaging-by-light-microscopy/equipment/software---fiji/). In some cases, bleed-through from the GFP signal into the CFP and RFP channel was detected. In these cases we processed the images as follows: 1) we calculated a normalization factor for the GFP channel = maximum GFP intensity / (maximum RFP intensity due to bleed-through – (minus) average RFP intensity in RGC layer); 2) we divided the GFP channel over this ratio generating a new image that corresponded to the bleed-through signal, 3) we subtracted this new image from the RFP channel. The same procedure was implemented for the CFP channel when necessary. In order to quantify the integrated fluorescence intensity from each layer, the boundaries of the region below the fluorescence curve corresponding to that layer were set to the point corresponding to the 50 % of the peak intensity (see Fig. 11b). The percentage of the signal corresponding to each cell type was calculated as the ratio between the area under the plot corresponding to each cell type layer and the area under the plot corresponding to that channel as a whole.

### Statistical analysis

In all cases, box plots represent the 25–75 % quartiles, the horizontal lines represent the median, and the short horizontal lines are the minimal and maximal values. All statistical analyses were performed as previously described [35, 36] using Past software [37]. As a routine, the datasets were checked for normality using Shapiro-Wilk normality test and for homogeneity of variances using Levene’s test. In the case of normal and homogeneous data, we performed a Student’s *t*-test for media comparison. If homogeneity requirement was not met, we performed a rank transformation [38]. In the case of non-normal data, we performed a Mann–Whitney test.

## Results

### Characterization of primary cilia in early stages of retina differentiation

**Fig. 1:**
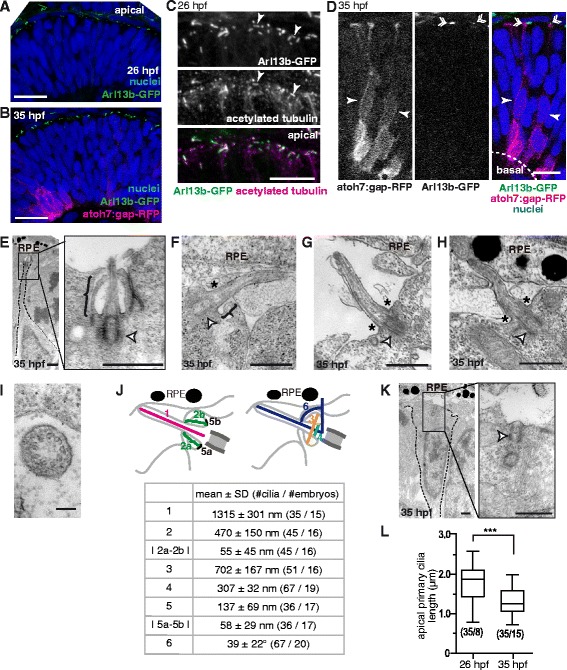
Main features of apical primary cilia in the early differentiating retinal neuroepithelium. The embryonic zebrafish retinal neuroepithelium was analyzed using confocal microscopy and TEM. **a**-**b** 26 hpf embryos expressing Arl13b-GFP (localized to primary cilia) (**a**) or double transgenic 35 hpf embryos expressing Arl13b-GFP and atoh7:gap-RFP (expressed in progenitors during the last cell cycle and in RGC neuroblasts) (**b**) were fixed and analyzed in toto using confocal microscopy. A 3D maximum intensity projection of a 3 μm-thick confocal stack is shown. **c** 26 hpf embryos expressing Arl13b-GFP were immunolabeled with anti-acetylated tubulin antibody. A maximum intensity projection of a 3 μm-thick stack of the apical region of the neuroepithelium is shown. The arrowheads show primary cilia with Arl13b-GFP and acetylated tubulin labeling. **d** Single confocal plane with a detail of the stack shown in **b**. It is possible to observe cells bearing a primary cilium (*double arrowhead*) and expressing low levels of gap-RFP (*full arrowheads*). **e**-**h** TEM micrographs showing examples of apical primary cilia, either with a complete (**e**, *bracket*), incomplete (**f**, *bracket - asterisk*) or absent ciliary pocket (**g** and **h**, *asterisks*). Primary cilia in close contact with RPE cells were also observed (**h**). The basal body is indicated with a white arrowhead. **​i** Cross section of an apically localized primary cilium. **j** Morphological parameters of apical primary cilia of 35 hpf embryos obtained from measurements performed on TEM micrographs. Measured features are summarized in the upper diagrams, and values (mean ± standard deviation) shown in the lower table. **k** TEM micrograph showing a basal body (white arrowhead) associated with the apical plasma membrane but lacking an axoneme. **l** Comparison of apically-localized primary cilia length at 26 and 35 hpf. The numbers in brackets represent the number of cilia / embryos measured in each case. (***) *p* < 0.001, Student’s *t* test. RPE: retinal pigment epithelium. Scale bars: A-B, 20 μm; C-D, 10 μm; E, 1 μm; F-H, 0.5 μm; I, 0.1 μm; K, 1 μm. For a high resolution image of Fig. 1 please see Additional file 12

We first analyzed the presence, localization and ultrastructure of primary cilia in the differentiating zebrafish neural retina by confocal and transmission electron microscopy (TEM) of 26 and 35 hpf (hours post-fertilization) embryos, just before and some time after RGC generation has started, respectively. We used double transgenic atoh7:gap-RFP/Arl13b-GFP (see Methods) embryos that allowed us to visualize the RGCs via a membrane form of mRFP under the control of an RGC progenitors/neuroblasts promoter, and cilia through the expression of Arl13b, a protein that localizes to the ciliary axoneme fused to GFP. Confocal analysis of Arl13b-GFP embryos at 26 hpf showed that all detectable cilia in the retinal neuroepithelium were localized apically (Fig. 1a), and that Arl13b-GFP signal coincided with the acetylated tubulin labeling, a general-use marker for cilia (Fig. 1c). As neurons start differentiating after 28 hpf, this latter marker loses its specificity, as tubulin acetylation extends to the whole cytoplasm of neuroblasts and neurons. Hence, all further description on cilia localization by confocal microscopy was achieved using Arl13b-GFP as a marker. At 35 hpf, when neurogenesis has spread through the ventronasal region of the retina, we observed that most cilia were still present at the apical border of the neuroepithelium (Fig. 1b). Some cells bearing an apical primary cilium also expressed low levels of the gap-RFP construct (Fig. 1d), suggesting that both cycling progenitors and early neuroblasts present an apically-localized primary cilium.

Likewise, our TEM analysis showed that in 35 hpf retinas most of the primary cilia are localized to the apical surface of the neuroepithelium, extending into the subretinal space (Fig. 1e-h). These cilia displayed different angles with respect to the neuroepithelial surface, albeit no correlation was evident between this angle and the region of the retina being examined. At the ultrastructural level, all these apical cilia had a 9 + 0 axoneme (Fig. 1i) and most (69/94) were immerse in a deep ciliary pocket (Fig. 1e), although in a few cases this structure was either incomplete (7/94; Fig. 1f) or absent (18/94; Fig. 1g-h). Some of the observed cilia projected into invaginations of retinal pigment epithelium (RPE) cells (Fig. 1h). Figure 1j presents a quantification of morphometric parameters of these apical cilia. Less frequently, we also observed basal bodies in close association with the plasma membrane but without a visible axoneme (Fig. 1k). In order to ascertain if these features were also present in a completely undifferentiated retinal neuroepithelium, we also analyzed apical primary cilia ultrastructure in 26 hpf embryos using TEM. Interestingly, we found that these cilia were morphologically similar to those from 35 hpf embryos, except for their length: cilia were significantly longer in 26 hpf retinas (Fig. 1l).

**Fig. 2:**
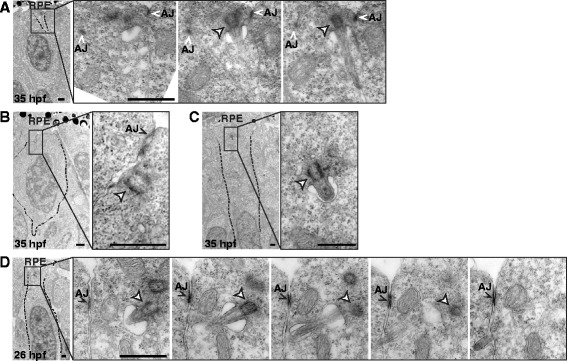
Primary cilia emerging from the basolateral membrane of retinal neuroepithelial cells. TEM micrographs of retinas from 26 and 35 hpf embryos. **a** Low magnification (*left*) and high magnification serial sections (*right*) of a retina from a 35 hpf embryo. A primary cilium emerges from the basolateral membrane, as evidenced by the presence of adherent junctions (AJ). **b** Basal body without an axoneme, but closely associated with the basolateral membrane. **c** Basal body with a short axoneme inside a cytoplasmic vesicle. **d** Low magnification (*left*) and high magnification serial sections (*right*) of a retina from a 26 hpf embryo showing a primary cilium emerging from the basolateral membrane. White arrowhead: basal body; RPE: retinal pigment epithelium. Scale bars: A-D, 1 μm. For a high resolution image of Fig. 2 please see Additional file 13

In addition to cilia emerging from the apical membrane, we found that around 10 % of total cilia in 35 hpf embryos (15/153; 23 embryos) were clearly located subapically, protruding from the basolateral cell membrane (Fig. 2a). Although their basal bodies were localized close to the apical border of neuroepithelial cells, these cilia emerged basally to the adherent junction belts and pointed towards the basal side of the neuroepithelium. We also observed some basal bodies docked to the basolateral membrane, without a visible axoneme (Fig. 2b) and small primary cilia inside cytoplasmic vesicles, with a short axoneme, pointing in a basal or basolateral direction (Fig. 2c). Basally oriented primary cilia have been previously observed in the mouse developing cortex, where they were present in neuroepithelial cells committed to delamination, so as to generate basal progenitors [39]. In the case of the neural retina, delamination only occurs in postmitotic cells, such as differentiating RGCs. To test if these retinal neuroblasts showed the same behavior as cortical progenitors, we analyzed 26 hpf embryos, in which progenitors have not yet become postmitotic. In these early embryos we also found a considerable number of basally oriented primary cilia (8 %, 10/123; 7 embryos; Fig. 2d).

### Cilia dynamics in early neuroblasts

**Fig. 3:**
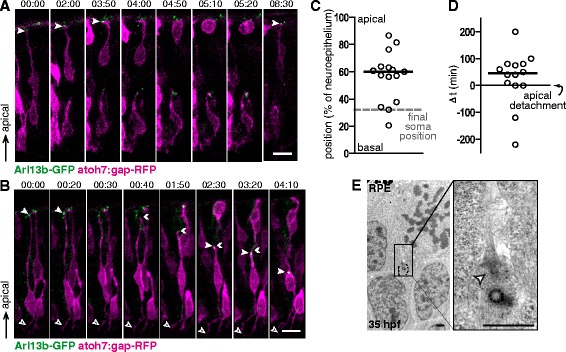
Primary cilia first appear in RGC neuroblasts during apical process retraction and in an apical position. **a**-**b** Blastomeres from double transgenic embryos (atoh7:gap-RFP/Arl13b-GFP) were transplanted into wild type hosts. The resulting embryos were imaged through time-lapse confocal microscopy from around 30 hpf onwards. Montages from 3D maximum intensity projections of the stacks are shown. **a** A progenitor is shown, which loses its primary cilium (*arrowhead*) at t = 4.00 h, previous to entering M phase. **b** Neuroblast imaged throughout apical process retraction. The full arrowhead denotes the presence and position of the primary cilium, while the double arrowhead denotes the apical tip of the retracting process and the empty arrowhead the region of axonal outgrowth. **c** Plot of the relative apico-basal position of the neuroepithelium in which primary cilia first appear during the retraction of the apical process. The final position occupied by the neuroblast cell bodies (*grey dashed line*) and the median value of the data (*black line*) are also shown. **d** Plot of the individual values and median time-delay (*black line*) between the initiation of apical retraction and primary cilia appearance. **e** TEM micrograph from a 35 hpf embryo, showing a primary cilium at the apical tip of a retracting process. The white arrowhead indicates the basal body. RPE: retinal pigment epithelium. Time is shown in hrs:min. Scale bars: A-B, 10 μm; E, 1 μm. For a high resolution image of Fig. 3 please see Additional file 14

To study the dynamics of primary cilia we performed live confocal microscopy on mosaic embryos resulting from the transplant of cells from double transgenic embryos (atoh7:gap-RFP/Arl13b-GFP) to unlabeled wild type embryos at the blastoderm stage. Transplanted embryos were imaged through time-lapse confocal microscopy from early developmental stages (30 hpf). In these movies, we were able to observe gap-RFP low-expressing cells bearing apically-localized Arl13b-labeled primary cilia. Some of these cells eventually divided, indicating that they were progenitors. As expected, dividing cells lost their cilia just before the onset of mitosis (Fig. 3a, Movie in Additional file 1).

**Fig. 4:**
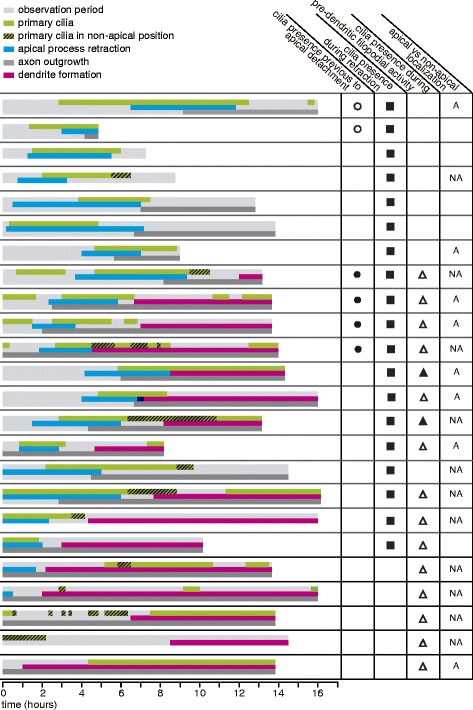
Primary cilia dynamics in relation to RGC differentiation events. Schematic representation and summary of the observations made in the time-lapse experiments. A single cell is represented in each row. For each cell, the presence of its primary cilium (*green*) is plotted in relation to the different events during RGC differentiation: apical process retraction (*cyan*), axon outgrowth (*dark grey*) and dendrite formation (*magenta*). The cells that show similar behaviors are indicated in the different columns: cells that show a primary cilium before apical detachment (●: cilium disappears previous to detachment; ○: cilium remains visible and moves basally previous to detachment); cells that show a primary cilium during the retraction of the apical process (■); cells that continuously show a primary cilium through the period from apical retraction to pre-dendritic filopodial activity/dendritogenesis (▲), and those in which the cilium disappears at least for a short time during this period (∆). The dashed regions in the diagrams indicate the time interval in which the primary cilium was localized to non-apical positions. Cells that showed this behavior are indicated as “NA”, and those in which the primary cilium remained apical after the retraction of the apical process are indicated as “A”. A total of 24 cells from 14 embryos in 12 different experiments were analyzed. For a high resolution image of Fig. 4 please see Additional file 15

After the last cell division, the neuroblast cell body moves towards the basal side of the neuroepithelium and the apical process detaches and begins to retract [8]. Some of the elongated atoh7-positive cells did not divide, but eventually retracted their apical processes and/or extended an axon on the basal surface, indicating that they were post-mitotic neuroblasts (Fig. 3b). In a few cases (6 cells) it was possible to observe an apically localized primary cilium previous to apical detachment. In four of these cells the primary cilium seemed to disappear before the onset of detachment (Fig. 3b; Movie in Additional file 2; Fig. 4, denoted as ●), while in the other two cells it did not disappear but moved basally, towards the cell body, before the beginning of the apical retraction process (Movie in Additional file 3; Fig. 4, denoted as ○). In these latter cases, the apical process detached and eventually reached the position of the cilium, which then regained an apical localization.

A primary cilium usually re-appeared during the retraction of the apical process, localizing to its tip, and remaining visible until the end of the retraction (Fig. 3b; Movie in Additional file 2; Fig. 4, denoted as ■). The position along the neuroepithelium where these primary cilia became evident again was variable among different cells, but most frequently we first detected the cilium in the central region of the neuroepithelium (i.e.,: halfway in the retraction process; Fig. 3c). The time of appearance of the primary cilium relative to the initiation of apical process retraction was also highly variable, although in most cases it happened within the following 1.5 h (Fig. 3d). Given the possibility that resolution constraints of our time-lapse experiments could hinder the visualization of cilia at early stages of retraction, we also analyzed the process by TEM. Cilia, both with and without a ciliary pocket, were observed on retracting apical processes either in close proximity to the apical surface of the neuroepithelium or in more basal positions, always pointing towards the apical surface (Fig. 3e). Importantly, this analysis allowed us to confirm the presence of apically localized primary cilia in retracting processes and showed that, at least in some cells, a primary cilium is present during the early stages of apical retraction. A summary of the behavior of all cells analyzed by time-lapse microscopy is shown in Fig. 4.

### Characterization of cilia in differentiating RGCs

**Fig. 5:**
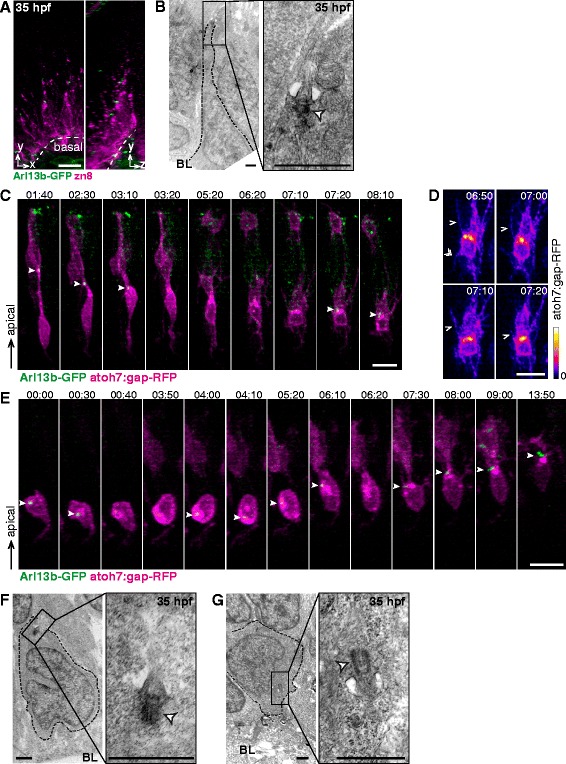
Upon completion of apical retraction, primary cilia may transiently disappear or lose their apical localization. **a** 3D maximum intensity projections of a region of the retina of a 35 hpf transgenic embryo expressing Arl13b-GFP and immunolabaled with zn8 antibody to identify RGCs. Primary cilia are observed colocalizing with the apical tip of cells finishing retraction. **b** TEM micrograph from a 35 hpf embryo showing a primary cilium at the tip of an apical process. The white arrowhead indicates the basal body. **c**-**e** Blastomeres from transgenic embryos (atoh7:gap-RFP/Arl13b-GFP) were transplanted into wild type hosts. The resulting embryos were imaged by time-lapse confocal microscopy from around 30 hpf onwards. Montages from 3D maximum intensity projections of the stacks are shown. (**d**) Detail of the cell shown in (**c**), where initial filopodial activity (*arrowheads*) previous to dendrite formation is observed; in order to highlight atoh7:gap-RFP signal intensity, a color ramp is used. The full white arrowheads indicate the presence and position of the primary cilium in **c** and **e**. **f**-**g** TEM micrographs from 35 hpf embryos, showing cells located in the basal region of the neuroepithelium (RGC neuroblasts) with primary cilia both in apical (**f**) and basal (**g**) positions. The white arrowhead indicates the basal body. BL: basal lamina. Scale bars: A, 10 μm; B, 1 μm; C-E, 10 μm; F-G, 1 μm. For a high resolution image of Fig. 5 please see Additional file 16

Towards the end of the apical process retraction, the differentiating RGC cell bodies have reached a basal position in the neuroepithelium. Once there, they generate an axon that grows adjacent to the basal lamina towards the optic nerve exit and dendritogenesis starts at the apical pole of these neuroblasts [8]. Early neuroblasts and RGCs express the surface adhesion protein Neurolin/DM-Grasp (labeled by the monoclonal antibodies zn5 or zn8), which can be used as a cell-type specific marker [40]. Arl13b-GFP embryos, fixed and labeled with either of these antibodies at 35 hpf, showed neuroblasts in the final stages of retraction with prominent cilia in an apical position (Fig. 5a). Cilia in similar positions were also observed in TEM micrographs (Fig. 5b). We further analyzed cilia dynamics at these later stages of neuronal differentiation in transplanted embryos using time-lapse confocal microscopy. We found that the primary cilium usually remained visible after apical retraction was completed, but it disappeared at least transiently during the initial steps of filopodial activity that leads to dendritogenesis (Fig. 5c, d; Movie in Additional file 4; Fig. 4, denoted as ∆) [41]. Only two cells showed a primary cilium throughout apical retraction and until the initiation of dendrite outgrowth (Fig. 4, denoted as ▲).

If we consider only those cells that showed a primary cilium after completion of apical process retraction and/or during the initial steps of dendrite formation, the primary cilium remained in the apical side of the cell during the imaging period in half of them (Fig. 4, denoted as “A”). Surprisingly, in the other half, primary cilia transiently lost their apical localization, occupying apparently random positions around the cell body, and rapidly moving from one position to another (Fig. 5e; Movie in Additional file 5; Fig. 4, denoted as “NA”). In TEM studies of 35 hpf embryos, fully formed cilia were rarely found in basally localized neuroblasts without an apical process. However, short cilia were eventually visualized, which exhibited different localizations and orientations around the cell (Fig. 5f and g). Interestingly, our time-lapse experiments showed that in cells bearing a primary cilium at the onset of dendritogenesis, the cilium was invariably localized to the base of the protrusion that finally branched to form the dendrites. This was the case even when cilia moved around the cell body before eventually reaching that position (Fig. 5e, Movie in Additional file 5).

**Fig. 6:**
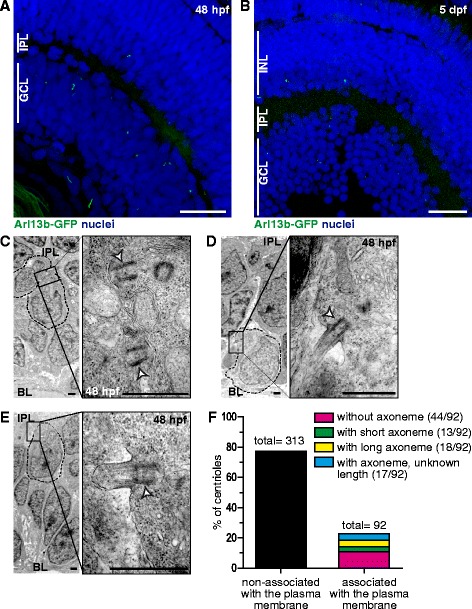
Primary cilia in maturing RGCs. **a**-**b** Arl13b-GFP transgenic embryos were fixed and imaged through confocal microscopy as whole-mounts (**a**, 48 hpf) or as cryosections (**b**, 5 dpf). Images shown are maximum intensity Z-projections of 1.5 μm (**a**) and 15 μm (**b**) confocal microscopy stacks. **c**-**e** TEM micrographs from retinas of 48 hpf embryos. In most of the cases we observed membrane-docked basal bodies without visible axonemes (**c**). Some cells showed basal bodies associated to long axonemes (**d**) or short axonemes with dilated tips (**e**). The white arrowhead indicates the basal body. **f** Comparison between the number of plasma membrane-associated and non-associated centrioles/centrosomes in the RGC layer of 48 hpf embryos (a total of 6 embryos were used in the quantification). The former were further classified in subtypes illustrated in the micrographs C, D and E. GCL: ganglion cell layer; IPL: inner plexiform layer; INL: inner nuclear layer; BL: basal lamina. Scale bars: A-B, 20 μm; C-E, 1 μm. For a high resolution image of Fig. 6 please see Additional file 17

By 48 hpf, all the RGCs in the central part of the retina have been born, their axons have exited the retina, and the synaptic connections have begun to form in the inner plexiform layer [42]. When we imaged Arl13b-GFP embryos fixed at 48 hpf, we were able to detect only a few primary cilia in the ganglion cell layer (Fig. 6a). A similar situation was observed at 5 days post fertilization (dpf) (Fig. 6b). After thorough TEM analysis of retinas from 48 hpf embryos, we observed a relatively small proportion of membrane-associated centrosomes in the ganglion cell layer (92/405, 22.7 %), of which nearly half (44/92, 48 %) did not nucleate visible axonemes, albeit they localized next to an electron-dense region of the plasma membrane (Fig. 6c and f). The rest of the centrosomes were acting as basal bodies of primary cilia of variable length. We found evident axonemes in only 19.5 % of the cases (18/92; Fig. 6d and f), and invariably, these cilia were positioned at the base of the RGC dendritic tree, where most were pointing basally. In 14 % of the cases (13/92) we could observe short cilia associated with basal bodies (Fig. 6e and f). These cilia presented dilated tips containing a granular, irregular material and without a visible axoneme or microtubules, although their proximal region appeared normal. The rest of the membrane-associated centrioles nucleated axonemes that could not be completely visualized in the section, and hence, of undefined length (17/92, 18.5 %) (Fig. 6f).

### Cilia dysfunction leads to early retinal differentiation defects

**Fig. 7:**
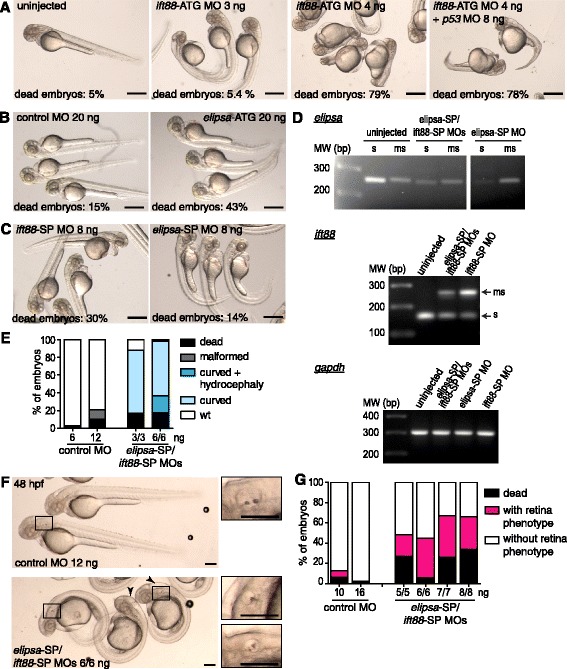
Evaluation of different morpholino oligomers for *elipsa* and *ift88* knock-down. **a**-**c** External phenotype of 48 hpf embryos injected with different morpholinos targeting the ciliary proteins *ift88* and *elipsa*; translational-blocking morpholinos: *ift88*-ATG MO (**a**), *elipsa-*ATG MO (**b**); splice-blocking morpholinos: *ift88*-SP or *elipsa*-SP (**c**). **d** RT-PCR analysis of *elipsa* and *ift88* mRNA levels in 35 hpf embryos either injected with *ift88*-SP (6 ng) or *elipsa*-SP (6 ng) alone or as a combination (6 ng each) (“s”: spliced mRNA form; “ms”: mis-spliced mRNA form). *Gapdh* mRNA was used as a control. **e** Evaluation of the external phenotype of 48 hpf embryos injected with different amounts of the combination of splice-blocking morpholinos (*elipsa*-SP/*ift88*-SP MOs), where “malformed” refers to morphological alterations not compatible with a classic “ciliary phenotype”. **f** Main characteristics of the external phenotype of double morphants at the dose used in the rest of the study. The black rectangle marks the position of the otic vesicle in the low magnification images, magnified in the insets. The arrowheads indicate embryos with enlarged brain ventricles. **g** Quantification of the percentage of atoh7:gap-GFP embryos injected with different amounts of control MO or *elipsa*-SP/*ift88*-SP MOs displaying reduced size of the RGC layer (“retina phenotype”), at 48 hpf. Scale bars: A-C, 500 μm; F, 200 μm. For a high resolution image of Fig. 7 please see Additional file 18

To evaluate the possible function of primary cilia during the early stages of retinal development and RGC differentiation, we tested different previously described morpholino oligomers (MOs), targeting ciliary-specific genes and known to generate typical ciliary phenotypes. A translation-blocking MO to *ift88* [28] was able to generate a recognizable but relatively weak ciliary phenotype at low doses (3 ng/embryo) in the genetic background of our fish lines. At slightly higher doses (4 ng/embryo), however, it caused generalized embryo mortality and severe defects that were not reverted by p53 MO co-injection (Fig. 7a). Another translation-blocking MO, in this case against *elipsa* [27], was not able to generate a marked ciliary phenotype, even at extremely high doses, such as 20 ng/embryo (Fig. 7b). Hence, we decided to design two splice-blocking MOs directed against these two genes (*ift88*-SP and *elipsa*-SP), with the intention of allowing the embryos to develop normally until gastrulation. Although the *ift88*-SP MO caused less embryo mortality or cell death than the translation-blocking one, even at 8 ng/embryo, phenotypes appeared too weak or absent (Fig. 7c). The *elipsa*-SP MO, on the other hand, produced very little embryo death and a recognizable ciliary phenotype, which was nevertheless still relatively weak (Fig. 7c). In spite of these results, both MOs were able to generate significant amounts of mis-spliced RNAs at the tested doses (6 ng each, Fig. 7d). Finally, a combination of these two splice-blocking MOs, at lower doses each, allowed us to obtain robust and reproducible ciliary phenotypes, with relatively little embryo or cell death (Fig. 7e). At 48 hpf, these double morphants (6 ng of each MO) displayed a ventrally-curved body and, in some cases, enlarged brain ventricles and abnormal otolith number (arrows and insets in Fig. 7f). This phenotype was reproduced in atoh7:gap-GFP transgenic embryos, where we observed an important reduction on the extent of the RGC layer at 48 hpf when compared to controls. We used this phenotypic feature to assay the ideal MO working doses for further experiments, confirming that a combination of 6/6 ng of the *elipsa*-SP/*ift88*-SP MOs gave the highest proportion of embryos with phenotype, while maintaining low levels of embryo death (Fig. 7g).

**Fig. 8:**
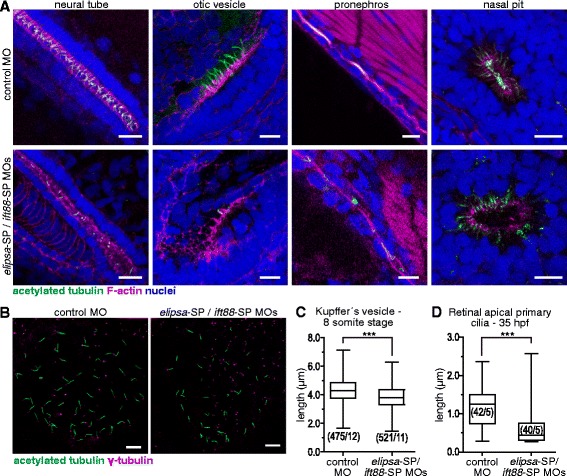
Effective reduction of primary cilia length in zebrafish embryos upon *elipsa* and *ift88* knock-down. **a** Confocal images of different ciliated organs from 48 hpf embryos. Cilia were labeled with an anti-acetylated tubulin antibody and F-actin with TRITC-phalloidin. **b** Kupffer’s vesicle of eight-somite stage embryos, where basal bodies were labeled with an anti-γ-tubulin antibody. **c** Comparison of primary ciliary length in Kupffer’s vesicle. The experiments were performed twice, with similar results; only the results from one of the experiments are shown. **d** Comparison of the length of apical primary cilia in the retina of 35 hpf morphant and control embryos. Measurements were made on TEM micrographs. In C and D the numbers in brackets represent the number of cilia and embryos analyzed in each condition. (***) *p* < 0.001, Mann–Whitney test. Scale bars: A-B, 10 μm. For a high resolution image of Fig. 8 please see Additional file 19

To further confirm the effectiveness of this MO combination, we examined cilia in different ciliated organs (neural tube, otic vesicle, pronephros and nasal pit) and, in all cases, found them to be reduced in number as well as shorter and more disorganized in morphant embryos in comparison to control embryos (Fig. 8a). We also quantified cilia length in the Kupffer’s vesicle at the eight-somite stage (around 12 hpf), observing a significant shortening of these organelles in morphant embryos (Fig. 8b and c). Finally, by using TEM, we evaluated the effect of the MOs in the differentiating neural retina, where we observed a significant reduction in apical primary cilia length (Fig. 8d). Overall, our results show that using a combination of *elipsa*-SP/*ift88*-SP MOs resulted in defective cilia formation and/or maintenance that led to well-established cilia-associated phenotypes while minimizing undesired effects, such as generalized cell death or gross developmental defects.

**Fig. 9:**
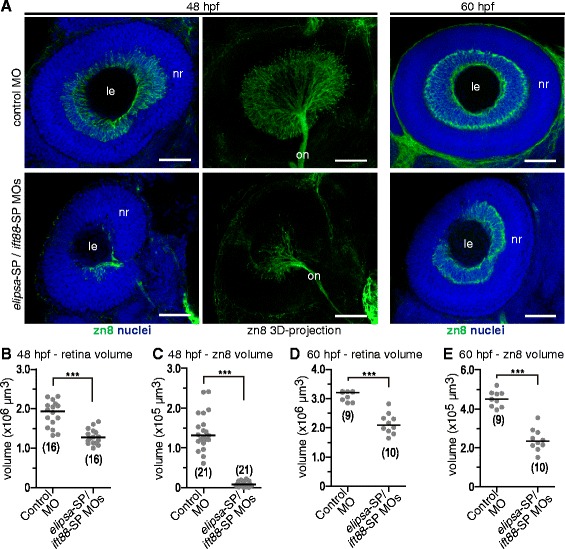
Embryos with impaired cilia have smaller eyes and a reduced RGC layer. **a** Confocal images of 48 hpf and 60 hpf retinas from embryos injected with control morpholino or *elipsa*-SP/*ift88*-SP MOs. RGCs were labeled with zn8 antibody. **b** and **d** Comparison of retinal volumes in 48 hpf (**b**) and 60 hpf (**d**) embryos. **c** and **e** Comparison of RGC layer volume (based on zn8 stain) in 48 hpf (**c**) and 60 hpf (**e**) embryos. The numbers in brackets represent the number of embryos quantified in each case (we quantified one eye per embryo). (***) *p* < 0.001. Comparisons were made using rank transformation and Student’s *t* test. nr: neural retina; le: lens; on: optic nerve. Scale bars: A, 50 μm. For a high resolution image of Fig. 9 please see Additional file 20

To further determine if primary cilia defects have an effect in early differentiation or organization of the retina, we injected wild type embryos with our combination of *elipsa*-SP/*ift88*-SP MOs, fixed them at 48 and 60 hpf and performed whole-mount immunofluorescence to analyze the retinal ganglion cell layer (using zn8 antibody) through confocal microscopy. As morphant embryos showed a notorious reduction in total eye size compared to controls (Fig. 9a), we quantified the volume of neural retinas on confocal stacks of 48 hpf embryos in both conditions. Double morphants showed a 38.4 % reduction in retinal volume when compared to controls (mean decrease from two independent experiments: 34.6 and 42.1 %; Fig. 9b). At this stage, and consistent with observations at low magnification described above, there was a pronounced reduction in the volume covered by zn8-positive RGCs: the ganglion cell layer appeared smaller and the optic nerve thinner in morphants compared to control embryos (Fig. 9a). We measured the volume spanned by the zn8 stain, excluding the extra-retinal tissues and the optic nerve. We observed a 95.4 % reduction in the volume of the zn8-positive retina in morphant embryos compared to controls (mean decrease from two independent experiments: 93.7 and 97.1 %; Fig. 9c). When we analyzed 60 hpf embryos we found that the RGCs had formed a complete layer (Fig. 9a). However, morphant embryos again showed a 26.8 % decrease in total retinal volume (mean decrease from two independent experiments: 18.7 and 35 %; Fig. 9d) and a 40.4 % reduction in zn8-positive retina compared to controls (mean decrease from two independent experiments: 32.6 and 48.1 %; Fig. 9e).

**Fig. 10:**
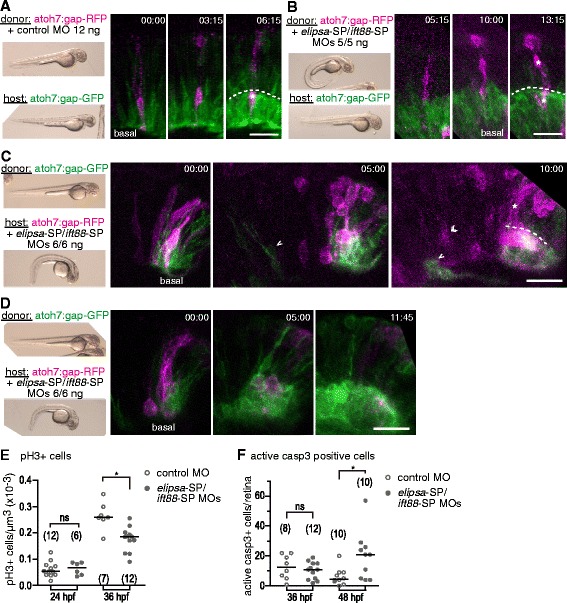
Cell-autonomous effect of cilia reduction on RGC differentiation. **a**-**b** Blastomeres from atoh7:gap-RFP embryos injected with control MO (12 ng) (**a**) or *elipsa*-SP/*ift88*-SP MOs (5/5 ng) (**b**) were transplanted into atoh7:gap-GFP hosts. The dotted line indicates de apical margin of the RGC layer of the host and the asterisk denotes the donor cells with high RFP expression. **c**-**d** Blastomeres from un-injected atoh7:gap-GFP embryos were transplanted into atoh7:gap-RFP hosts injected with *elipsa*-SP/*ift88*-SP MOs (6/6 ng). Retinas with small (**c**) and large (**d**) clones of GFP positive cells were observed. In C the dotted line indicates de apical margin of the RGC layer formed by the donor GFP positive cells, and the asterisk denotes host cells with high RFP expression. Single arrowheads mark GFP expressing cells that appear before RFP expressing cells (*double arrowheads*). In all the cases (**a**-**d**) the resulting host embryos were imaged by time-lapse confocal microscopy beginning at around 30 hpf. Fluorescence images show montages from 3D maximum intensity projections of the stacks from three different time points. On the left, the external phenotype at 48 hpf of the donor and host embryos is shown in each case. Time is expressed in hrs:min. Scale bars: A-D, 20 μm. **e** Comparison of the number of mitotic cells (identified by anti-pHistone H3 labeling) in the ventro-nasal region of 24 and 36 hpf retinas, expressed as the number of positive cells per unit of volume. (*) *p* = 0.0014. **f** Comparison of the number of apoptotic cells (identified by anti-activated Caspase 3 labeling) per retina in 36 and 48 hpf embryos. (*) *p* = 0.009. All experiments were performed twice, with similar results; only the results from one of the experiments are shown. The numbers in brackets represent the number of embryos quantified in each case (we quantified one eye per embryo). (ns) non-significant difference. Comparisons were made using rank transformation and Student’s *t* test. For a high resolution image of Fig. 10 please see Additional file 21

In order to ascertain if these observed early defects in RGC layer formation were due to a cell-autonomous effect of cilia reduction in RGC progenitors or neuroblasts, instead of an indirect effect caused by a perturbed retinal environment, we performed blastomere transplantation experiments between atoh7:gap-RFP embryos, either injected with control or *elipsa*-SP/*ift88*-SP MOs, and atoh7:gap-GFP untreated embryos, followed by in vivo time-lapse observation (Fig. 10a and b; Movies in Additional files 6, 7 and 8). In 5/5 embryos observed, control MO-treated RFP-positive cells were able to differentiate apparently normally in the untreated atoh7:gap-GFP retina, dividing and differentiating into RGCs or other cell types in the same way and timing as the surrounding GFP-positive cells (Fig. 10a; Movie in Additional files 6 and 7). After several attempts of transplanting double morphant (6/6 ng of each MO) cells into untreated embryos, we failed to detect transplanted cells expressing atoh7:gap-RFP in the retina by 32 hpf. As this could be due to a reduced capacity of the cilia-impaired cells to compete in the wild-type environment, we lowered the MO dose to 5 ng each. Under these conditions, we could detect few embryos in which small clones (1–3 cells) of atoh7:gap-RFP cells appeared in the atoh7:gap-GFP wild-type retina. In 3/5 of the in vivo imaged embryos, the observed gap-RFP-positive cells behaved in an apparently normal way when compared to the surrounding gap-GFP cells, while in 2/5 cases, three clones included some cells that appeared either delayed in differentiation or remaining in the outer retinal layers, in spite of expressing a relatively high level of gap-RFP (asterisk, Fig. 10b; Movie in Additional file 8). We then performed the converse transplantation experiment, from untreated atoh7:gap-GFP embryos into *elipsa*-SP/*ift88*-SP morphant atoh7:gap-RFP embryos. Here, transplantation was more efficient, and two types of behavior could be observed in the transplanted cells. In 4/7 cases, small clones of GFP-positive cells were able to quickly differentiate into RGCs, inserting in the RGC layer and extending an axon, in most cases ahead of the surrounding morphant RFP-positive cells (Fig. 10c; Movie in Additional file 9). In 2/7 cases, however, large clones expressing GFP rapidly proliferated and started to differentiate as RGCs, while very few RFP-positive host cells could be detected (Fig. 10d; Movie in Additional file 10).

We reasoned that the decrease in retina and ganglion cell layer volume, like the apparent delay in RGC differentiation, could be due either to reduced cell proliferation, to increased cell death, or a combination of both. To discriminate between these possibilities we performed whole-mount immunofluorescence against phosphorylated Histone H3 (pH3), present in mitotic cells from late prophase to the end of telophase, and for activated Caspase 3, indicative of early stages of apoptosis. We then quantified pH3 positive cells in the ventro-nasal region of the retina and the total number of activated Caspase 3-positive cells per eye, in embryos of different developmental stages. Regarding cell proliferation, while we did not observe differences at 24 hpf, our results showed a significant decrease in mitotic index in morphant embryos at 36 hpf (Fig. 10e). In the case of cell death, we observed an increase in apoptotic nuclei at 48 hpf while not at an earlier stage (36 hpf; Fig. 10f).

**Fig. 11:**
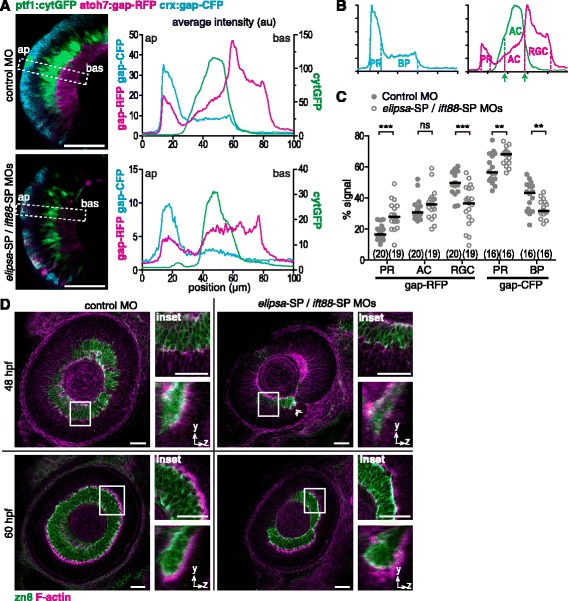
Cilia impaired embryos show a preferential delay in RGCs formation. **a** Left: average intensity Z-projections of 10 μm-thick confocal stacks of the nasal region of retinas from 48 hpf SoFa1 embryos injected with control MO or *elipsa*-SP/*ift88*-SP MOs. The dotted line delimits the area used to measure the fluorescence intensity profile from apical to basal, plotted in the right side for each image. ap: apical; bas: basal; au: arbitrary units. **b** Diagram indicating the regions in the SoFa1 retina fluorescence profile plots that were selected to determine the relative contribution of each cell type to the total signal in RFP and CFP channels (the GFP signal was only used to determine the extension spanned by amacrine cells in the RFP channel). The boundaries of each region were set at the distance corresponding to 50 % of the maximum fluorescence for each peak. **c** Plot of the percentage of signal intensity in the gap-RFP and gap-CFP channels corresponding to each cell type in embryos injected with control MO or *elipsa*-SP/*ift88*-SP MOs. The number of embryos analyzed in each case is shown in brackets. (***) *p* < 0.001, (**) *p* < 0.01, ns: non-significant difference. Comparisons were made using rank transformation and Student’s *t* test. PR: photoreceptors; AC: amacrine cells; RGC: retinal ganglion cells; BP: bipolar cells. **d** Confocal images of retinas from wild-type embryos injected with control MO or *elipsa*-SP/*ift88*-SP MOs and fixed at 48 and 60 hpf. The boxed regions were magnified in the upper insets, while lower images are orthogonal 3D projections of these insets. The white arrowhead indicates a patch of IPL in the region adjacent to the optic nerve exit. Scale bars: A, 50 μm; D, 30 μm. For a high resolution image of Fig. 11 please see Additional file 22

Altogether, these results suggest that the integrity of primary cilia is essential at the progenitor cell level, and therefore other retinal cell types might also be affected. To evaluate this possibility, we used the triple transgenic zebrafish line SoFa1, which allows for the individualization of the different retinal cell types through the expression of atoh7:gap-RFP (RGCs, amacrine cells, horizontal cells and cone photoreceptors), ptf1a:cytGFP (amacrine and horizontal cells), crx:gap-CFP (cone photoreceptors and bipolar cells) [26]. We injected these embryos with control morpholino or *elipsa*-SP/*ift88*-SP MOs and analyzed them using in vivo time-lapse microscopy. We observed a general decrease in the signal in morphant embryos in comparison to controls, consistent with reduced progenitor cell proliferation (Fig. 11a). We also analyzed the fluorescence profile for each channel along a rectangular selection perpendicular to the plane of the retina (Fig. 11a; Movie in Additional file 11). In control embryos, RGCs (labeled by gap-RFP alone) were the first cells to become evident, closely followed in time by cone photoreceptors (gap-RFP / gap-CFP) and amacrine cells (cytGFP / gap-RFP). By the end of the time-lapse acquisition (around 48 hpf) horizontal cells (cytGFP / gap-RFP) and bipolar cells (gap-CFP) started to become evident. In morphant embryos injected with *elipsa*-SP/*ift88*-SP MOs, the gap-RFP signal corresponding to RGCs was more evenly distributed along the retina and was usually defined later than the peaks corresponding to the other cell types (Movie in Additional file 11). To quantify this observation, we determined the relative contribution of each cell type to the total signal in the last frame of these movies and in fixed 48 hpf morphant embryos (Fig. 11a, b and c). Interestingly, the proportion of gap-RFP signal corresponding to RGCs was significantly reduced in morphant embryos in comparison to controls, with a concomitant increase in the signal corresponding to photoreceptors but not amacrine cells. A similar comparison made on gap-CFP-positive cells also showed a slight, albeit significant, increase of cone photoreceptors relative to bipolar cells (cells that are born late, and hence probably more affected by the general cell differentiation delay at 48 hpf).

To further confirm the possibility that RGC generation and/or differentiation is preferentially reduced after primary cilia impairment, we analyzed the extension of the inner plexiform layer (IPL) through whole-mount staining with TRITC-conjugated phalloidin (Fig. 11d). Embryos injected with control morpholino and fixed at 48 hpf presented a thin IPL layer only in the ventro-nasal region of the retina, while at 60 hpf the IPL was thicker and extended throughout the whole neural retina. Embryos injected with *elipsa*-SP/*ift88*-SP MOs and fixed at 48 hpf had a smaller patch of IPL restricted to the region adjacent to the optic nerve exit, where the first RGCs differentiate (Fig. 11d, arrowhead). At 60 hpf, the IPL of morphant embryos had extended throughout the retina, but appeared thinner and more disorganized than in control embryos (Fig. 11d, insets).

## Discussion

### Dynamic apical cilia in the early neurogenic retinal neuroepithelium

Pioneering electron microscopy studies showed, many decades ago, the presence and apparent dynamics of primary cilia in the undifferentiated and differentiating neuroepithelium, both in the neural tube and the retina [19, 20]. In recent years, the finding that primary cilia act as cellular “antennae” has renewed the interest of developmental neurobiologists in understanding the possible roles of these organelles in neurogenesis and neuronal differentiation. Here, we sought to characterize the behavior and possible functions of primary cilia in these processes, starting by assessing their presence and localization in the embryonic zebrafish retina before and around the initial stages of neurogenesis. It must be noted that because of the morphological features of primary cilia, particularly their small size and the fact that there is only one per cell, added to their functional properties (cilia are constantly disassembled and reassembled along the cell cycle), it is simply not possible to assure that a cell that does not display a detectable cilium is actually a non-ciliated cell. We observed the presence of numerous primary cilia in the early retina, both before and shortly after the onset of neurogenesis, indicating that probably most retinal neuroepithelial cells are ciliated. These cilia mostly localized apically, and were pointing towards the sub-retinal space. In addition, most of them presented a deep ciliary pocket, a structure that has been documented in different cell types both in vitro and in vivo (reviewed in [43]). Some early studies suggested a link between the ciliary pocket and different stages of ciliation, as well as the possibility of different pathways in cilia formation. More recently however, it has been shown that the ciliary pocket is a site of active endocytosis [44, 45]. Interestingly, its presence, size and morphology were extremely variable among retinal neuroepithelial cells, which may therefore suggest differences in their cellular activity.

The accumulation of centrosomes at the apical side of different neuroepithelia has been extensively documented, and even used as a cell polarity marker [39, 46]. In the present work, we observed that many of these apical centrosomes are actually primary cilia basal bodies. However, we also found a few cases of apically localized centrosomes that were not nucleating an axoneme (although they usually appeared docked at the plasma membrane), as well as short primary cilia inside cytoplasmic vesicles. These possibly represented different stages of cilia formation [47], even though we cannot distinguish if these cells are cycling progenitors or neuroblasts. Our in vivo experiments showed that both situations are possible: while there is a cilia cycle related to the cell cycle (i.e.,: in progenitor cells), cilia in postmitotic neuroblasts may also be highly dynamic. We found that neuroepithelial cells committed to exit the cell cycle (evidenced by the expression of atoh7:gap-RFP), presented a primary cilium until a short time before mitosis. Accordingly, studies in chick neural tube have shown that primary cilia are lost during mid-G2 in order for the centrosome to engage in mitosis [48]. Regarding postmitotic cells, our in vivo experiments interestingly showed that some of these cells had visible primary cilia while still attached to the apical border, although these organelles were transiently lost in many cells around the period of detachment (see below).

We also found primary cilia that, albeit being localized to the apical region of neuroepithelial cells, emerged from a basal position with respect to adherens junctions. These cilia may be translocated or re-formed at the basolateral surface and the cilia that were observed inside cytoplasmic vesicles may correspond to intermediate stages of any of these processes. This observation is highly reminiscent of that reported in the mouse cortex where cells committed to delamination in the embryonic telencephalon present basolaterally localized primary cilia, the proportion of which increases at the onset of neurogenesis [39]. In the zebrafish retina, we found the proportion of basolateral primary cilia to be the same at 26 and 35 hpf (stages before and after neurogenesis initiation, respectively), whereas ciliary length showed a decrease at the latter stage. Thus, cilia shortening might present a stronger correlation with neurogenesis in the zebrafish retina than basolateral cilia localization.

### Cilia dynamics in differentiating RGCs

**Fig. 12:**
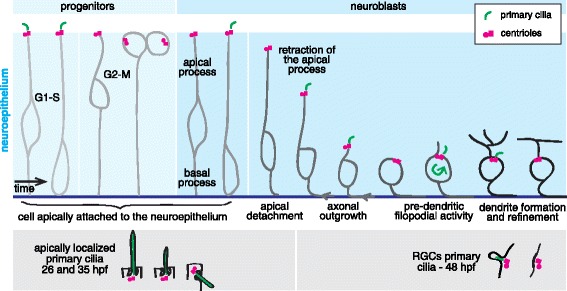
Primary cilia dynamics in progenitors and differentiating RGC neuroblasts. In this diagram we summarize some of the main features of primary cilia behavior during early stages of retinal differentiation, based both on live fluorescence images of atoh7:gap-RFP/Arl13b-GFP expressing cells and on electron microscopy images. In the lower part of the figure the main ultrastructural characteristics found in apically localized primary cilia in early stages of development and of RGCs primary cilia by 48 hpf are also outlined. For a high resolution image of Fig. 12 please see Additional file 23

Our findings on the presence and dynamics of primary cilia in early stages of RGC differentiation are summarized in Fig. 12. In the zebrafish, recently born RGC neuroblasts undergo a transition from a neuroepithelial into a neuronal type of polarity. It was shown that these neuroblasts must detach their apical process from apical N-Cadherin-based adhesions, and that during the initial stages of retraction, different apical markers remain accumulated at the tip of the apical process [8, 49]. This includes the centrosome, which was observed to remain apical during apical process retraction and until after axon outgrowth. The centrosome acts in many cases as a basal body to a primary cilium. In chick and mouse spinal cord neuroblasts it was shown that the cilium, along with some other apical components, is shed from the tip of the apical process during detachment, while the centrosome remains in the retracting apical process [50]. On the other hand, Spear and colleagues, also studying the chick neural tube, have reported that neuroblasts undergoing delamination maintain their apical primary cilium [48]. Our in vivo studies using the same cilia marker as these two previous studies (Arl13b-GFP) showed that even if most RGC neuroblasts lack a visible primary cilium around the moment of apical detachment, in some cells the primary cilium accompanied the retraction of the apical process from the beginning. Probably due to our imaging conditions, we failed to visualize cilia from neuroblasts remaining at the apical border upon detachment. Hence, we cannot rule out the possibility that there is apical shedding during zebrafish RGC apical retraction, and our data suggest that even if either shedding or resorption of primary cilia may occur, they are not an absolute prerequisite for delamination.

The appearance of the primary cilium during apical process retraction was highly variable both in time and position across the neuroepithelium, as was the period it remained visible (see Fig. 4). However, in all cells, the cilium remained apical throughout retraction, in accordance to previous work that reported an apically localized centrosome in RGCs all through retraction [8] and at the initial stages of dendrite formation [2]. Consistently, apically-localized primary cilia in differentiating RGCs had also been reported in early electron microscopy studies of the mouse retina [20]. Surprisingly, however, we also observed a highly dynamic primary cilium regarding localization around the cell body surface, from the end of retraction to just before the initiation of dendrite formation. These movements are fast (can be observed in 10 min intervals), and might be related to the necessary cellular rearrangements that occur during the period between axon and dendrite formation. Interestingly, these movements ceased at the onset of dendritogenesis, with the primary cilium localizing to the base of the growing dendrites. Similar movements of the centrosome of differentiating RGCs, although in a more prolonged period of time, were observed when knocking-down Laminin α1, an essential signal for neuronal orientation in the zebrafish retina [2]. In tangentially migrating cortical interneurons, highly dynamic primary cilia that change in length and position during migration have been shown to be involved in sensing environmental Shh, and possibly other signaling molecules [17, 18]. Cortical neuroblasts form primary cilia postnatally, at a stage when their migration has ended [51]. Likewise, dentate granule cells born in adult mice, form primary cilia after reaching their final positions in the hippocampus, around the time of dendrite formation and synaptic connection establishment [16]. It has been shown that in these systems the primary cilium, which localizes to the base of the apical dendrite, is necessary for dendrite refinement and synapse formation [16, 52].

### RGCs are preferentially affected by cilia dysfunction during retinal development

The particular localization and dynamics of cilia in differentiating RGCs made us wonder what roles the organelle might have in the generation or differentiation of these neurons. As a first approach to evaluate the physiological role of cilia in the developing retina, we opted for a knockdown strategy using morpholino oligomers. IFT88 and Elipsa are two ciliary proteins that have been shown to directly interact and to be essential for intraflagellar transport in the zebrafish, whose mutants and knockdowns have been reported to give clear and reproducible “ciliary phenotypes” [27, 28]. In our hands, the best results were obtained with a combination of MOs against these two genes, as we reasoned that through this approach we were going to maximize the chances of observing a ciliary phenotype with relatively low doses of individual MOs, avoiding cilia-independent alterations. In addition, we used splice-blocking MOs to avoid affecting early developmental processes. This combination of MOs effectively reduced cilia number and length in different organs and tissues, and gave a clearer ciliary phenotype than the individual MOs used at higher doses.

Previous work by others aimed at characterizing the in vivo functional role of genes involved in ciliogenesis in the zebrafish, such as *elipsa*, *ift88*, *ift57* or *ift172* [27, 28, 53, 54], showed, by analyzing single-mutants for these genes, that the major phenotype in the retina was the cell-autonomous progressive loss of photoreceptor cells, evident from 3 dpf onwards. Here, we also observed very little or no early retinal phenotype after the individual injection of MOs to *ift88* and *elipsa*. Double-morphants, however, showed a significant and sustained reduction in retinal volume from early stages of differentiation, which correlated with both a decrease in the number of mitosis and an increase in cell death throughout the retina. The experiments on SoFa1 embryos also showed decreased numbers of all retinal cell types. This effect of cilia impairment on cell proliferation/survival was cell-autonomous, as can be concluded from the blastomere transplantation experiments. Interestingly these experiments indicated that MO-treated retinal cells had an extremely reduced capacity to compete against their neighboring cells, as: 1) very scarce transplanted morphant atoh7:gap-RFP cells could be detected in wild-type host embryos, and 2) wild-type transplanted atoh7:gap-GFP cells tended to appear earlier than, and in several cases overcame, the host atoh7:gap-RFP cells. Taken together, these results suggest that primary cilia are functional at the progenitor cell level, regulating mitosis and possibly cell cycle exit.

Interestingly, while we observed a 38 % reduction in total retina volume, the volume of the RGC layer transiently decreased up to 95 %. This effect was more evident at 48 hpf than at later stages, suggesting a partial recovery of neuronal differentiation as development advanced. In addition, the 48 hpf morphant RGC layer appeared to be in earlier developmental stages when compared to controls and the inner plexiform layer was only visible in morphant embryos at the anterior-ventral part of the retina, where cell differentiation begins [55], while it was complete in controls. Consistent with this supposition, we observed that transplanted wild-type atoh7:gap-GFP cells tended to differentiate much earlier than the surrounding atoh7:gap-RFP cells in morphant hosts. In vivo experiments in the SoFa1 fish line further indicated that the delay in neuronal generation was more prominent in RGCs than in other cell types. A proportional increase in the photoreceptor layer was also noticed, indicating that cell fate decisions were affected. A causal relationship between cell cycle regulation and neuronal cell fate choice has been reported in different regions of the central nervous system [56]. Therefore, it could be possible that an altered cell cycle progression upon cilia disruption could account for the observed reduction in RGC number with respect to photoreceptors. Indeed, the manipulation of the timing of cell cycle exit in *Xenopus* retinal progenitors affected the generation of early neuronal cell types (as RGCs) at the expense of late-generated neurons (as bipolar cells) [57].

## Conclusions

We have shown here that relatively short primary cilia are present in neural progenitors and early neuroblasts of the neural retina in the zebrafish. The most remarkable features of these cilia are that they tend to remain localized to the apical region of the cells, and that they become extremely dynamic particularly during neuroblast polarity transitions, such as apical detachment and between axon and dendrite formation. Finally, our cilia disruption experiments, knocking-down *elipsa* and *ift88*, underscore a cell-autonomous role for cilia at cell proliferation and survival, as well as in neuronal cell-type specification.

## Ethics approval and consent to participate

All manipulations described including zebrafish have been approved by the local Animal Ethics Committee at Institut Pasteur de Montevideo (CEUA, reg. n° 010 / 2013) and by the Uruguayan National Animal Ethics Committee (CNEA, reg. n° 002/11).

## Consent for publication

Not applicable.

## Additional files

Additional file 1: Time-lapse video corresponding to montage in Fig. 3a. A 3D maximum intensity projection of a confocal stack over time is shown. A progenitor (cell body marked with an asterisk) expressing gap-RFP (magenta) is observed previous to and during mitosis. It is possible to observe a primary cilium (pc) some time before cell rounding and its reappearance after division. Time is expressed in hrs:min. (MOV 8409 kb) Additional file 2: Time-lapse video corresponding to montage in Fig. 3b. A 3D projection of a confocal stack over time is shown. In this movie a neuroblast (cell body marked with an asterisk) is shown previous to detachment from the apical region of the neuroepithelium. This cell has already started an axonal outgrowth (ax) at the beginning of the acquisition. It is possible to observe the presence of a primary cilium (pc) during retraction of the apical process (ap) and during the initial stages of dendritogenesis. Time is expressed in hrs:min. (MOV 10497 kb) Additional file 3: Time-lapse video showing a cell (asterisk) with a primary cilium which moves basally previous to apical process detachment. A 3D projection of a confocal stack over time is shown. In the right panel only the Arl13b-GFP channel is shown, while in the left panel the merge with the gap-RFP channel is shown. The presence and localization of the primary cilium (pc) is indicated, as well as the tip of the apical process (ap) during retraction. Axonal outgrowth is also indicated (ax). Time is expressed in hrs:min. (MOV 8895 kb) Additional file 4: Time-lapse video corresponding to montage in Fig. 5c. A 3D projection of a confocal stack over time is shown. It is possible to observe a neuroblast (cell body marked with an asterisk) after the initiation of apical process detachment through the initial stages of dendritogenesis. This cell has already started an axonal outgrowth (ax) at the beginning of the movie. The presence and localization of a primary cilium is indicated with an arrowhead (pc). Time is expressed in hrs:min. (MOV 3697 kb) Additional file 5: Time-lapse video corresponding to montage in Fig. 5e. A 3D projection of a confocal stack over time is shown. It is possible to observe a basally localized neuroblast (asterisk), which has already extended an axon (ax), through the initial stages of dendritogenesis. The presence and localization of a primary cilium (pc) is indicated with an arrowhead. Time is expressed in hrs:min. (MOV 6377 kb) Additional file 6: Time-lapse video corresponding to montage in Fig. 10a. In this time-lapse it is possible to observe a small clone of cells expressing atoh7:gap-RFP from a control MO (12 ng) injected embryo. Host cells express atoh7:gap-GFP. A 3D projection of a confocal stack over time is shown. Time is expressed in hrs:min. (MOV 5787 kb) Additional file 7: Time-lapse video showing the retina of an embryo resulting from the transplant of blastomeres from control MO injected donor embryos to un-injected hosts. In this time-lapse it is possible to observe a large number of cells expressing atoh7:gap-RFP from a control MO (12 ng) injected embryo. Host cells express atoh7:gap-GFP. A maximum intensity projection of the stack is shown. Time is expressed in hrs:min. (MOV 4363 kb) Additional file 8: Time-lapse video corresponding to montage in Fig. 10b. In this time-lapse it is possible to observe a few cells expressing atoh7:gap-RFP from an embryo injected with *elipsa*-SP/*ift88*-SP MOs (5/5 ng). Host cells express atoh7:gap-GFP. A 3D projection of a confocal stack over time is shown. Time is expressed in hrs:min. (MOV 5303 kb) Additional file 9: Time-lapse video corresponding to montage in Fig. 10c. In this time-lapse it is possible to observe a small number of cells expressing atoh7:gap-GFP, which develop in the retina of an embryo injected with *elipsa*-SP/*ift88*-SP MOs (6/6 ng). Host cells express atoh7:gap-RFP. A 3D projection of a confocal stack over time is shown. Time is expressed in hrs:min. (MOV 3796 kb) Additional file 10: Time-lapse video corresponding to montage in Fig. 10d. In this time-lapse it is possible to observe a large clone of cells expressing atoh7:gap-GFP, which develop in the retina of an embryo injected with *elipsa*-SP/*ift88*-SP MOs (6/6 ng). Host cells express atoh7:gap-RFP. A 3D projection of a confocal stack over time is shown. Time is expressed in hrs:min. (MOV 3850 kb) Additional file 11: Time-lapse video and fluorescence profile analysis of SoFa1 embryos. Embryos were injected with control MO (right panels) or *elipsa*-SP/*ift88*-SP MOs (left panels) and imaged in vivo from 30 hpf onwards. A region in the ventro-nasal retina is shown in the upper panels and its corresponding fluorescent intensity profile over time in the lower panels. Each time frame corresponds to 10 min. (MOV 7625 kb) Additional file 12: Main features of apical primary cilia in the early differentiating retinal neuroepithelium. (PDF 2898 kb) Additional file 13: Primary cilia emerging from the basolateral membrane of retinal neuroepithelial cells. (PDF 1690 kb) Additional file 14: Primary cilia first appear in RGC neuroblasts during apical process retraction and in an apical position. (PDF 3553 kb) Additional file 15: Primary cilia dynamics in relation to RGC differentiation events. (PDF 109 kb) Additional file 16: Upon completion of apical retraction, primary cilia may transiently disappear or lose their apical localization. (PDF 6861 kb) Additional file 17: Primary cilia in maturing RGCs. (PDF 3994 kb) Additional file 18: Evaluation of different morpholino oligomers for elipsa and ift88 knock-down. (PDF 3369 kb) Additional file 19: Effective reduction of primary cilia length in zebrafish embryos upon elipsa and ift88 knock-down. (PDF 4352 kb) Additional file 20: Embryos with impaired cilia have smaller eyes and a reduced RGC layer. (PDF 2212 kb) Additional file 21: Cell-autonomous effect of cilia reduction on RGC differentiation. (PDF 3922 kb) Additional file 22: Cilia impaired embryos show a preferential delay in RGCs formation. (PDF 4731 kb) Additional file 23: Primary cilia dynamics in progenitors and differentiating RGC neuroblasts. (PDF 118 kb)

## Abbreviations

dpf: days post-fertilization
hpf: hours post-fertilization
MO: morpholino oligomer
RGC: retinal ganglion cell
RPE: retinal pigment epithelium
TEM: transmission electron microscopy
