# Supplementary figures and images for: Characterization of primary cilia during the differentiation of retinal ganglion cells in the zebrafish
Source: Neural Dev. 2016 Apr 6;11:10. doi: 10.1186/s13064-016-0064-z (PMC4823885; doi:10.1186/s13064-016-0064-z)

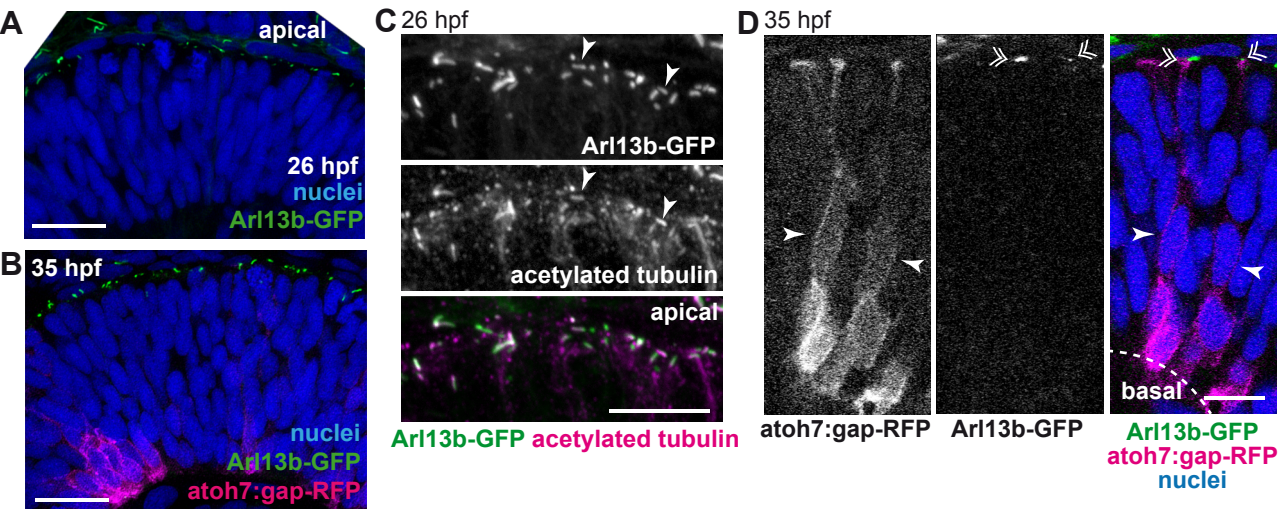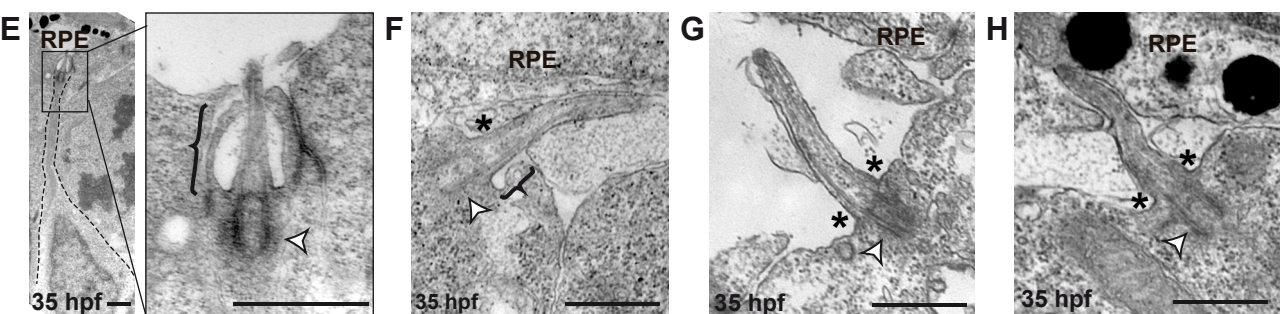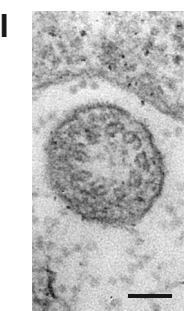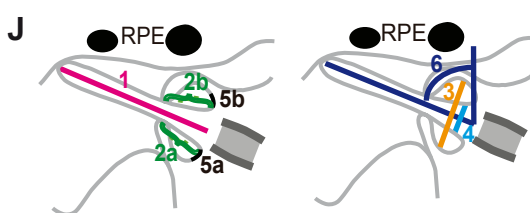

|       | mean $\pm$ SD (#cilia / #embryos) |
|-------|-----------------------------------|
| 1     | 1315 $\pm$ 301 nm (35 / 15)       |
| 2     | 470 $\pm$ 150 nm (45 / 16)        |
| 2a-2b | 55 $\pm$ 45 nm (45 / 16)          |
| 3     | 702 $\pm$ 167 nm (51 / 16)        |
| 4     | 307 $\pm$ 32 nm (67 / 19)         |
| 5     | 137 $\pm$ 69 nm (36 / 17)         |
| 5a-5b | 58 $\pm$ 29 nm (36 / 17)          |
| 6     | 39 $\pm$ 22° (67 / 20)            |

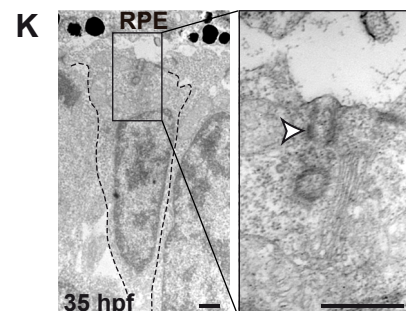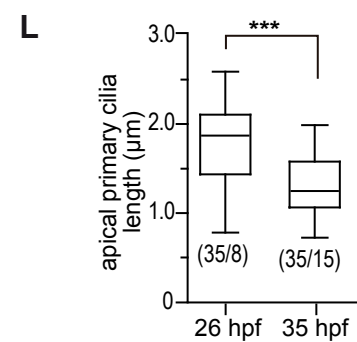

Supplement: Additional file 12: — Main features of apical primary cilia in the early differentiating retinal neuroepithelium. (PDF 2898 kb) [file 13064_2016_64_MOESM12_ESM.pdf]

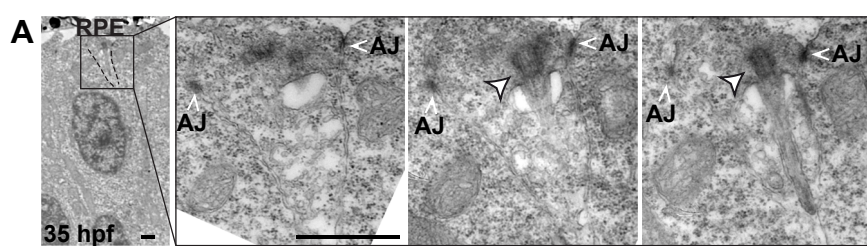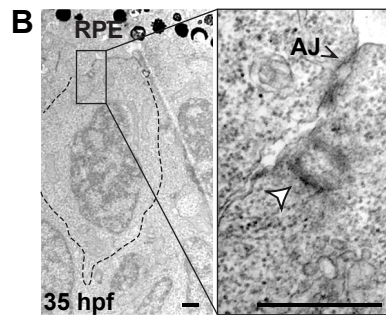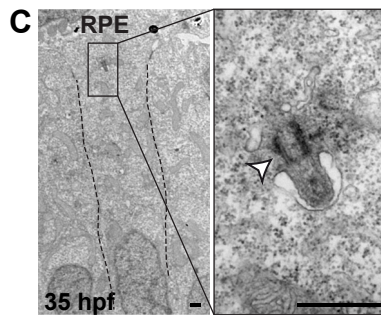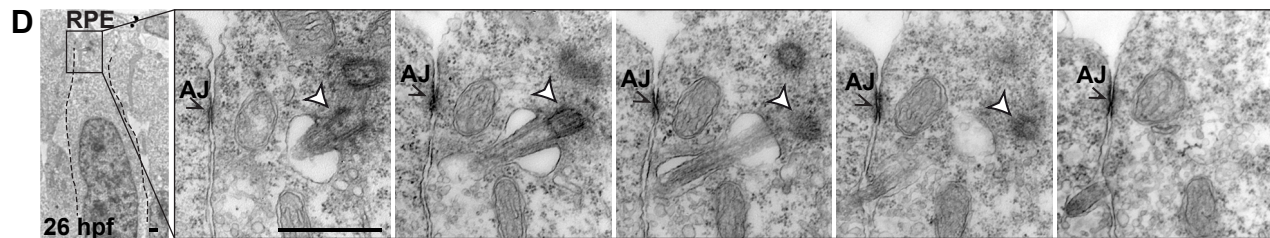

Supplement: Additional file 13: — Primary cilia emerging from the basolateral membrane of retinal neuroepithelial cells. (PDF 1690 kb) [file 13064_2016_64_MOESM13_ESM.pdf]

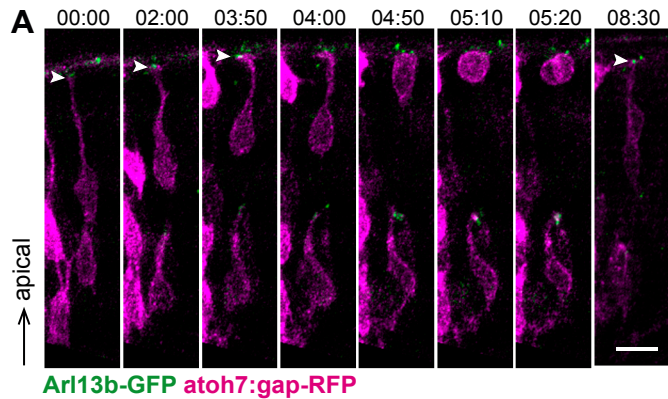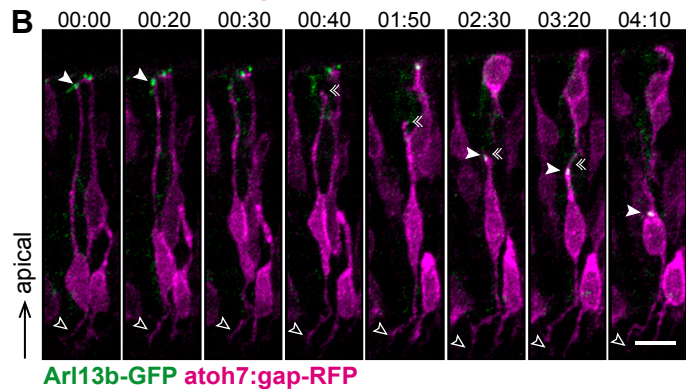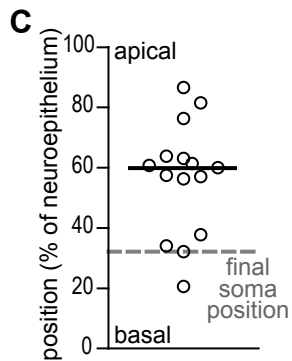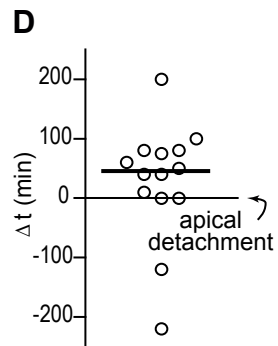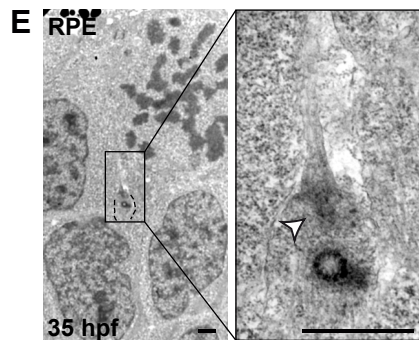

Supplement: Additional file 14: — Primary cilia first appear in RGC neuroblasts during apical process retraction and in an apical position. (PDF 3553 kb) [file 13064_2016_64_MOESM14_ESM.pdf]

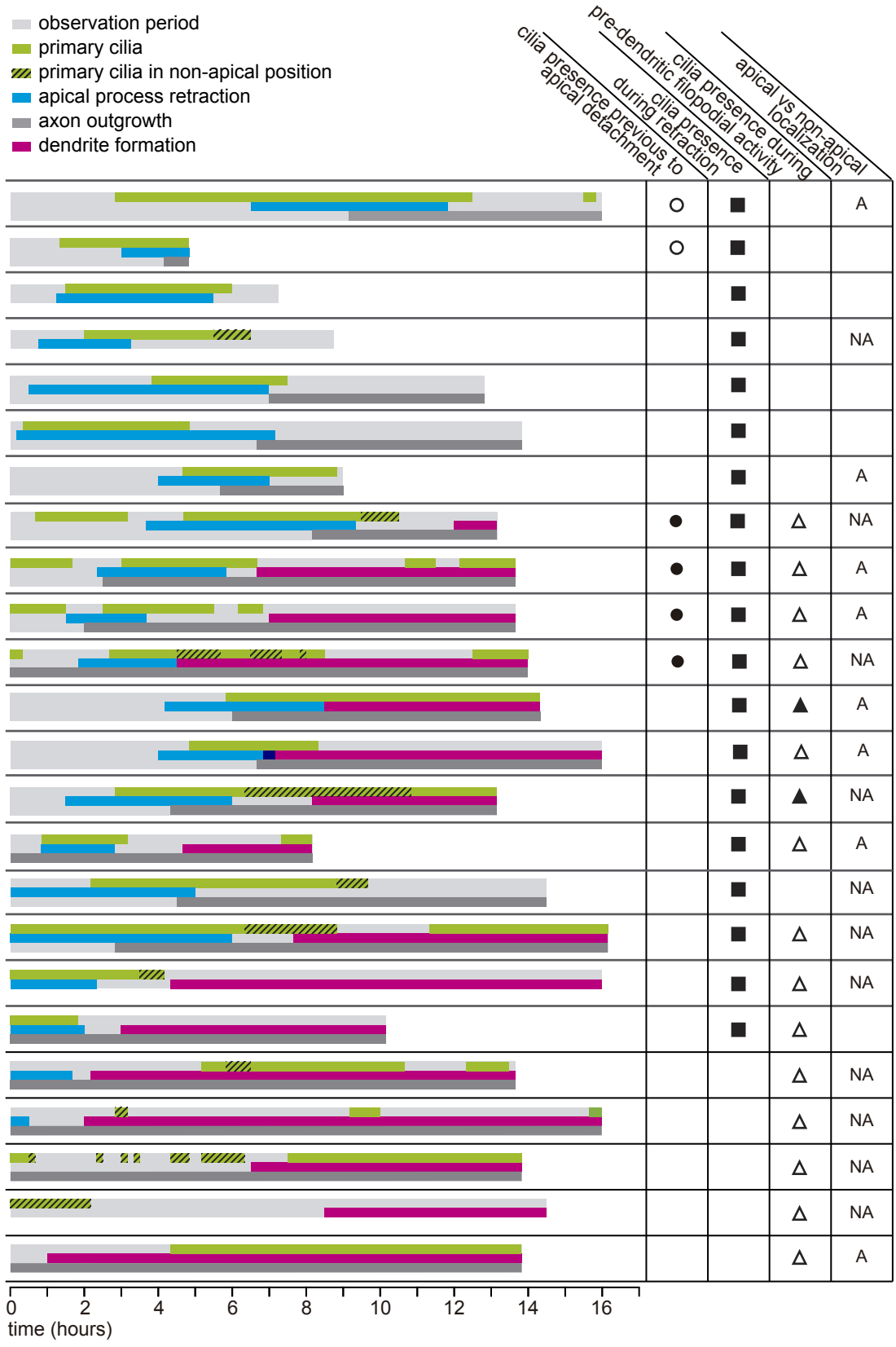

Supplement: Additional file 15: — Primary cilia dynamics in relation to RGC differentiation events. (PDF 109 kb) [file 13064_2016_64_MOESM15_ESM.pdf]

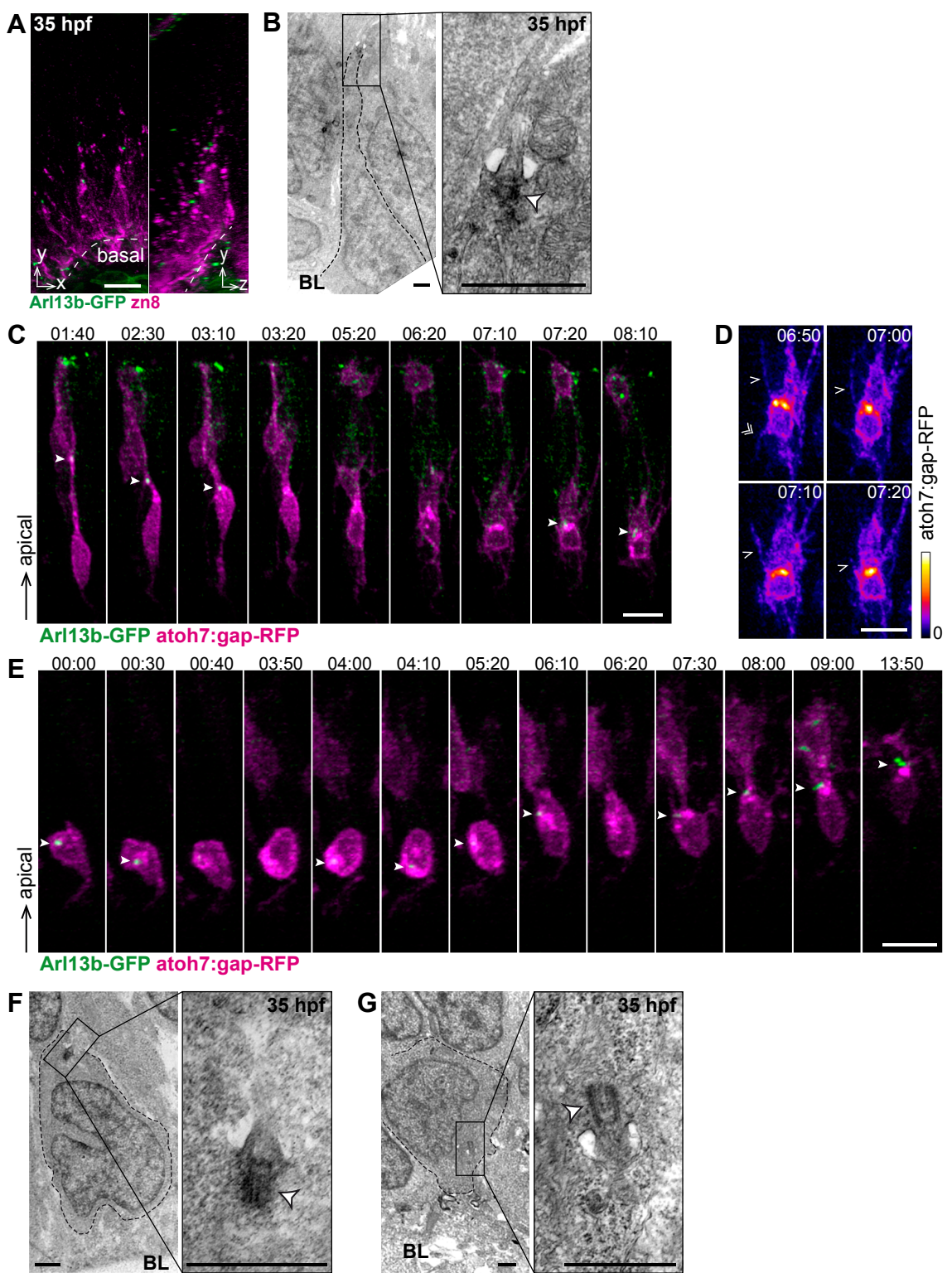

Supplement: Additional file 16: — Upon completion of apical retraction, primary cilia may transiently disappear or lose their apical localization. (PDF 6861 kb) [file 13064_2016_64_MOESM16_ESM.pdf]

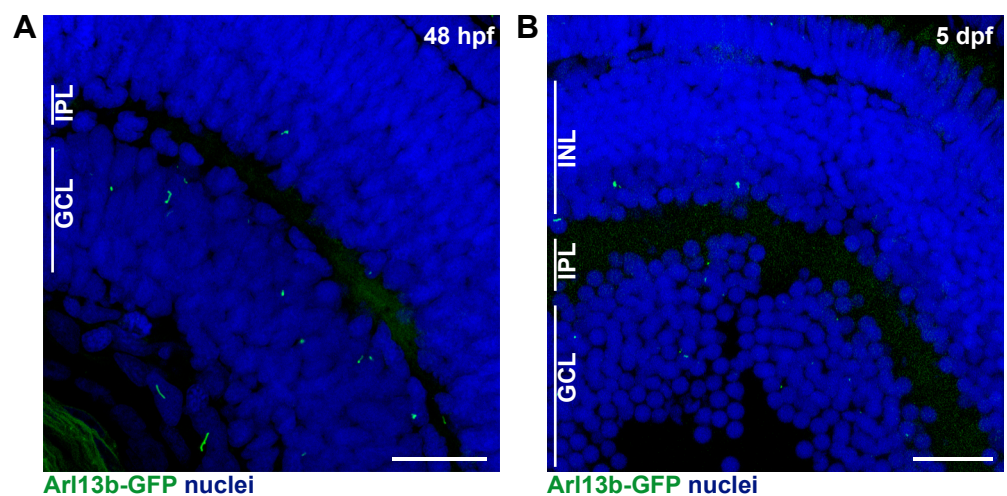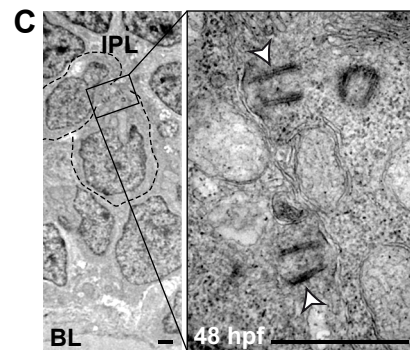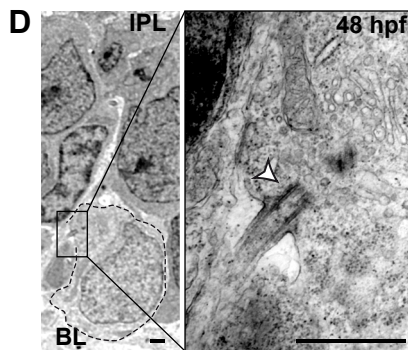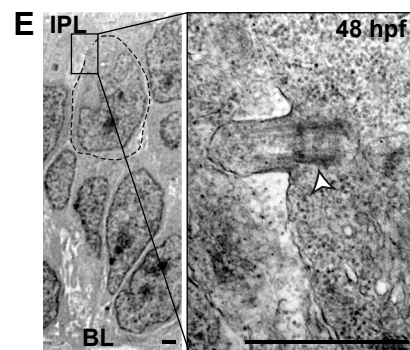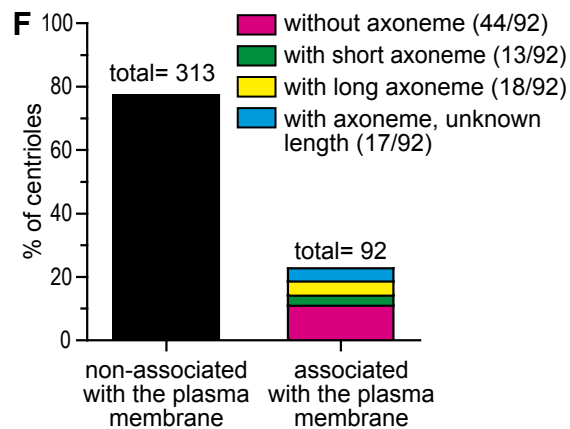

Supplement: Additional file 17: — Primary cilia in maturing RGCs. (PDF 3994 kb) [file 13064_2016_64_MOESM17_ESM.pdf]

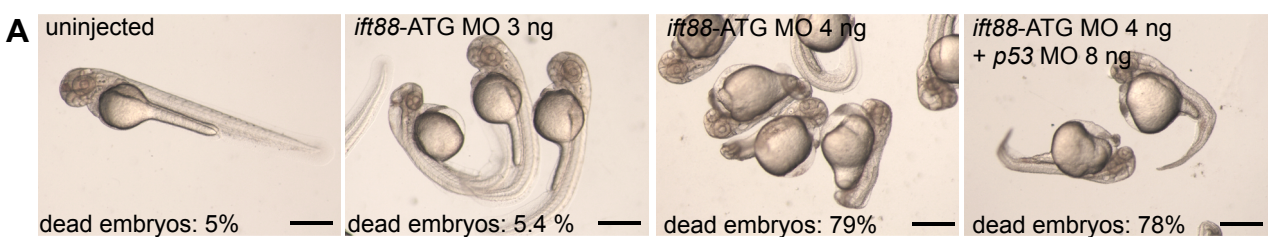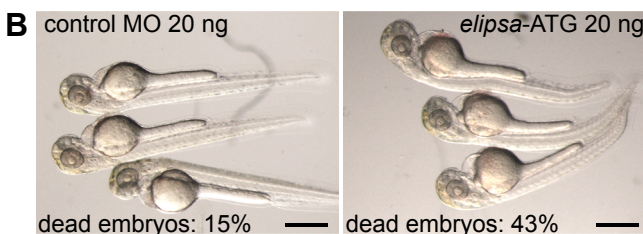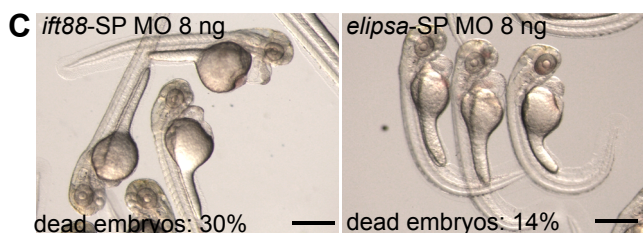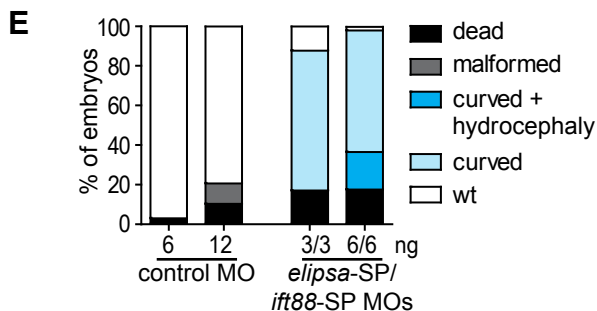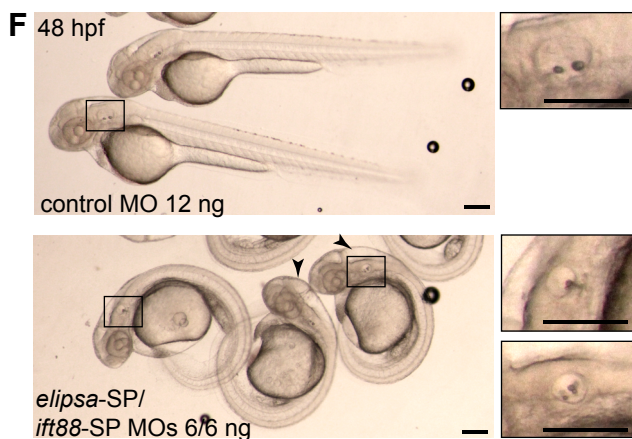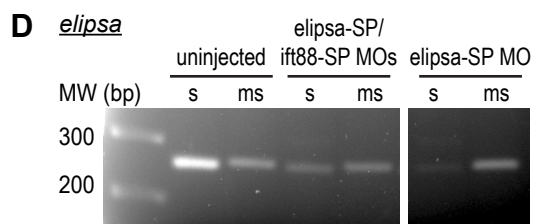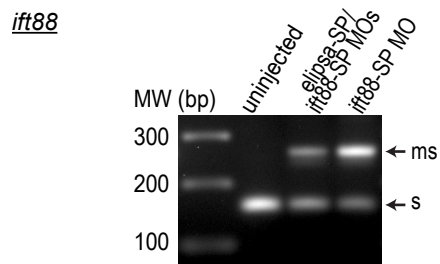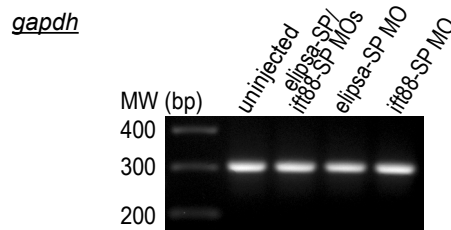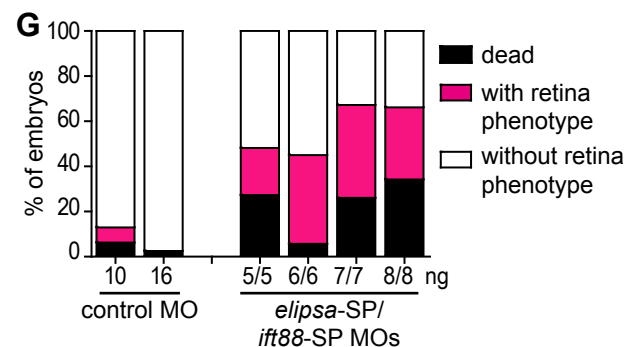

Supplement: Additional file 18: — Evaluation of different morpholino oligomers for elipsa and ift88 knock-down. (PDF 3369 kb) [file 13064_2016_64_MOESM18_ESM.pdf]

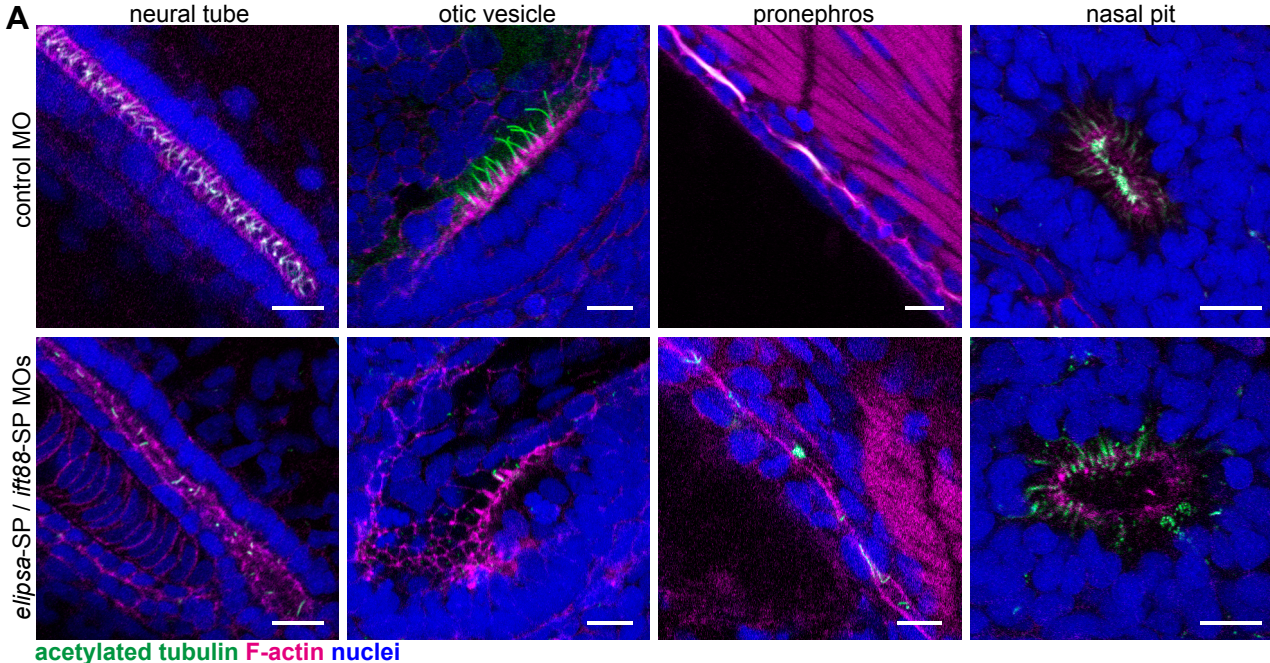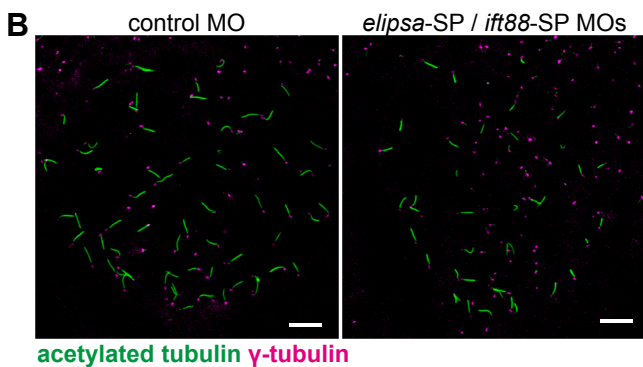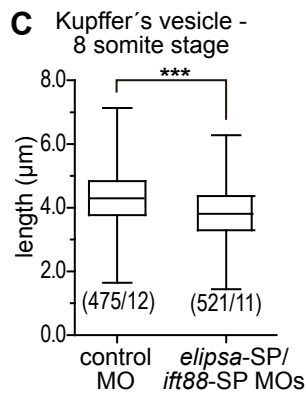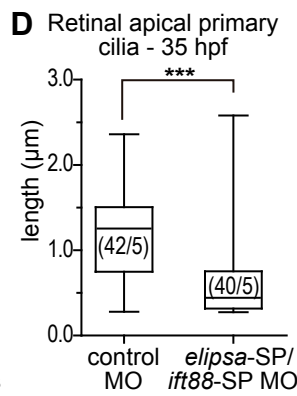

Supplement: Additional file 19: — Effective reduction of primary cilia length in zebrafish embryos upon elipsa and ift88 knock-down. (PDF 4352 kb) [file 13064_2016_64_MOESM19_ESM.pdf]

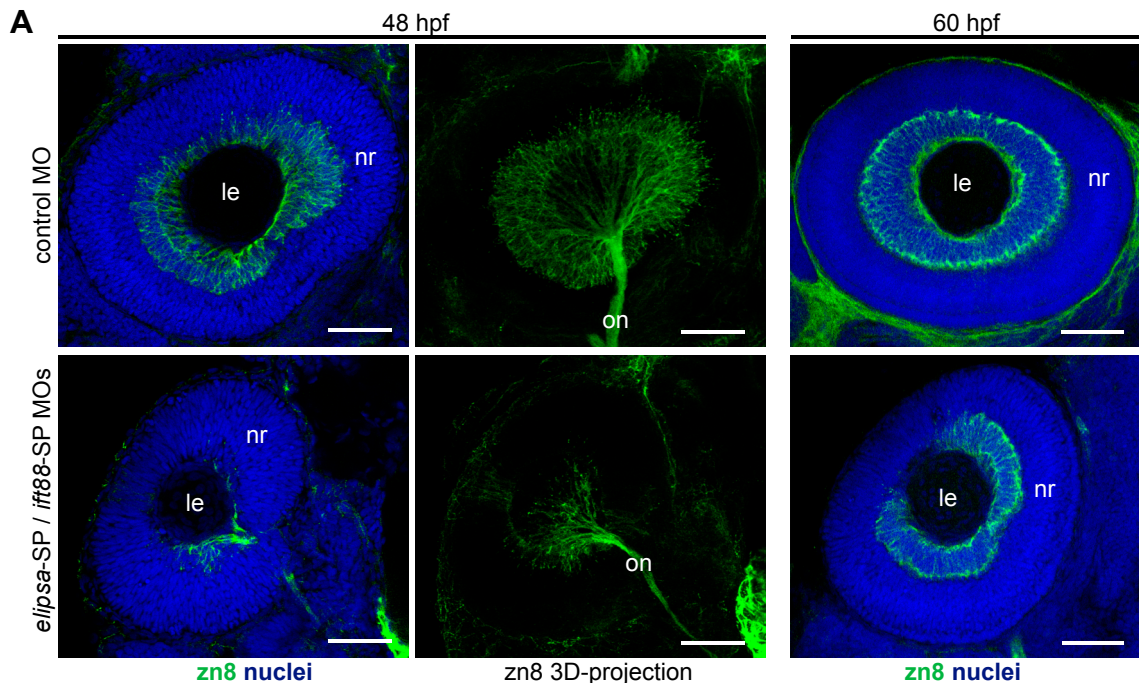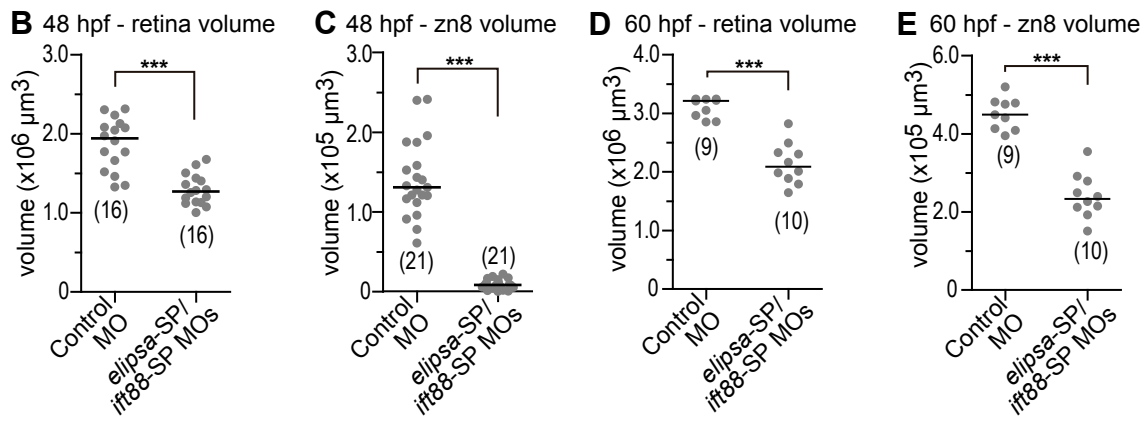

Supplement: Additional file 20: — Embryos with impaired cilia have smaller eyes and a reduced RGC layer. (PDF 2212 kb) [file 13064_2016_64_MOESM20_ESM.pdf]

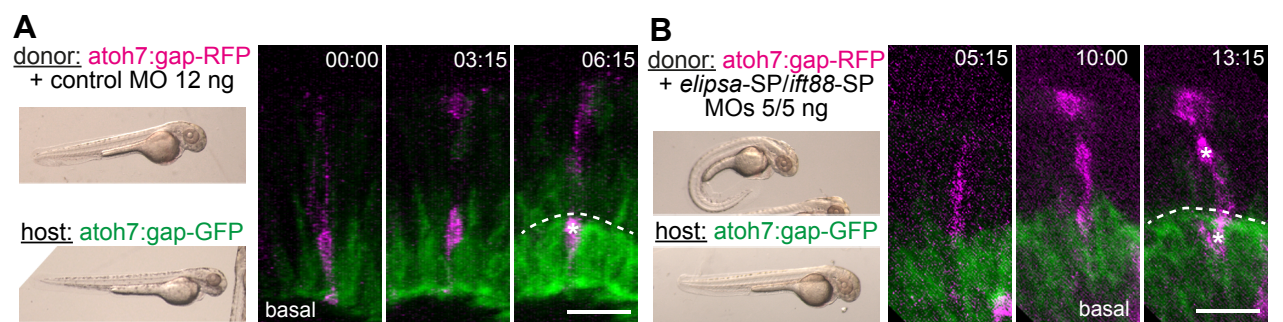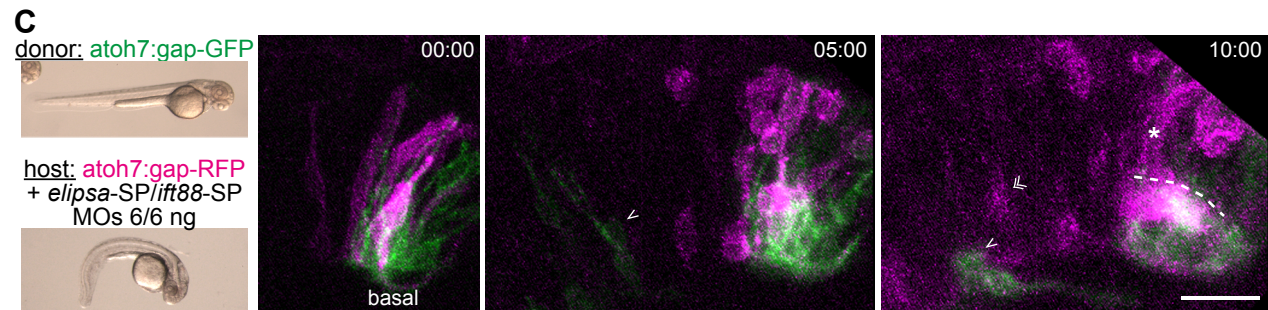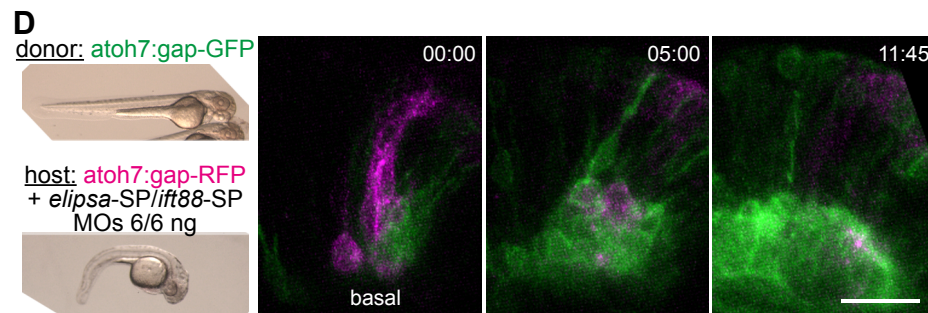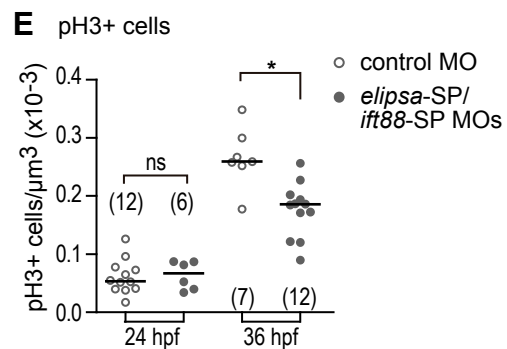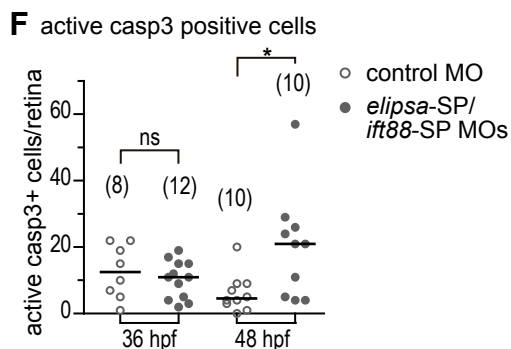

Supplement: Additional file 21: — Cell-autonomous effect of cilia reduction on RGC differentiation. (PDF 3922 kb) [file 13064_2016_64_MOESM21_ESM.pdf]

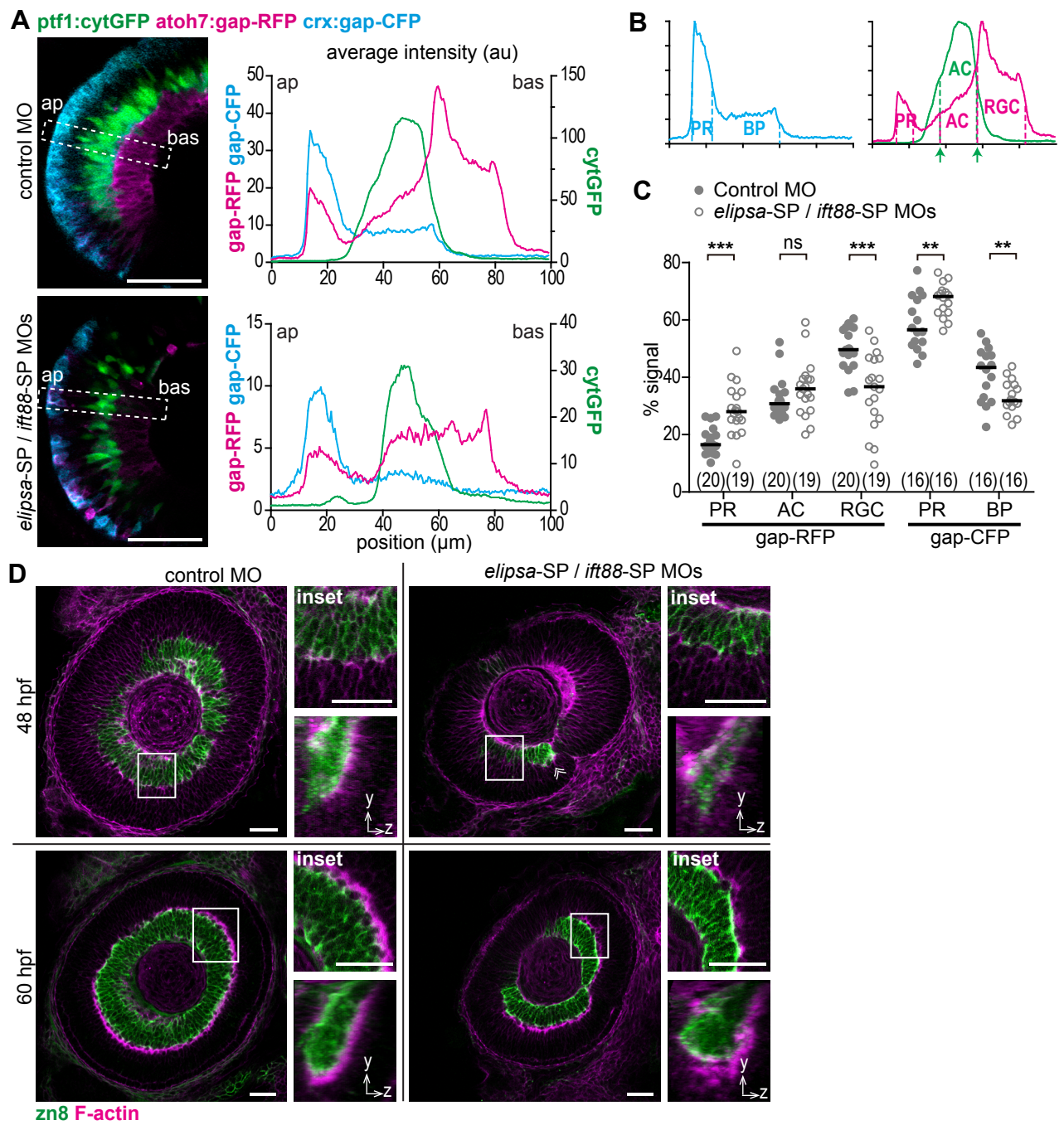

Supplement: Additional file 22: — Cilia impaired embryos show a preferential delay in RGCs formation. (PDF 4731 kb) [file 13064_2016_64_MOESM22_ESM.pdf]
