# Supplementary material for: Characterization of primary cilia during the differentiation of retinal ganglion cells in the zebrafish
Source: Neural Dev. 2016 Apr 6;11:10. doi: 10.1186/s13064-016-0064-z (PMC4823885; doi:10.1186/s13064-016-0064-z)
Supplement: Additional file 23: — Primary cilia dynamics in progenitors and differentiating RGC neuroblasts. (PDF 118 kb) [file 13064_2016_64_MOESM23_ESM.pdf]

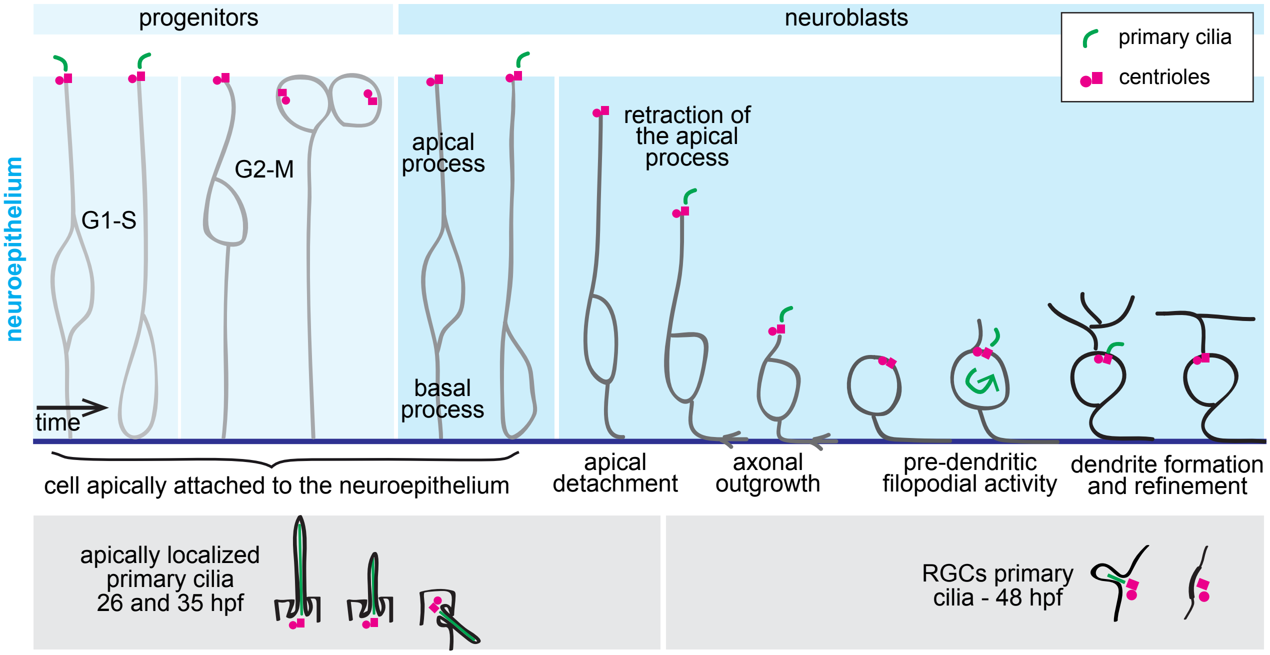
progenitors
neuroblasts
primary cilia
centrioles
neuroepithelium
G1-S
G2-M
apical process
basal process
time
retraction of the apical process
cell apically attached to the neuroepithelium
apical detachment
axonal outgrowth
pre-dendritic filopodial activity
dendrite formation and refinement
apically localized primary cilia 26 and 35 hpf
RGCs primary cilia - 48 hpf
